# Functional and structural impacts of oncogenic missense variants on human polo-like kinase 1 protein

**DOI:** 10.3389/fbinf.2025.1680578

**Published:** 2025-12-02

**Authors:** Gayatri Munieswaran, Venkatraman Manickam

**Affiliations:** School of Bioscience and Technology, Vellore Institute of Technology, Vellore, Tamil Nadu, India

**Keywords:** plk1, nsSNPs, cancer, biomarker, molecular dynamic simulation

## Abstract

**Introduction:**

The polo-like kinase 1 (PLK1), a master key mitotic regulator, is frequently expressed in various types of cancers and associated with poor prognosis. The missense mutations in PLK1 may compromise its structural integrity and functional interactions, contributing to tumorigenesis.

**Methods:**

This study utilized a comprehensive computational pipeline to identify deleterious missense variants across multiple cancers. 207 non-synonymous single nucleotide polymorphisms (nsSNPs) were retrieved from cBioPortal, and 11 high-risk variants were prioritized using functional and structural prediction tools, such as SIFT, PolyPhen-2, I-mutant 2.0, and so on. Prognostic prevalence was evaluated via Kaplan-Meier survival analysis, and functional networks were explored using STRING. The structural dynamics of modeled mutations were analyzed through molecular dynamic simulations over 100 ns.

**Results:**

The kinase domain mutations such as L244F, R293C, and R293H and polo-box domain mutations such as A520T were found to cause deviations in structural stability, flexibility, solvent exposure, and compactness compared to wild-type. Further, PLK1 overexpression correlated with poor overall survival of patient outcomes in many types of cancers, including breast, liver, lung, kidney, and pancreatic cancers. Protein-protein interaction revealed PLK1’s involvement in oncogenic pathways.

**Discussion:**

The study highlights the structural and functional implications of oncogenic PLK1 mutations, emphasizing their role in cancer progression. Integrating predictive and dynamic exploration approaches facilitates prioritization of variants with potential clinical relevance.

**Conclusion:**

The nsSNPs in PLK1 may perturb conformational stability and functions of the protein. Further experimental validation and discovery of novel inhibitors might develop mutation-specific interventions in precision oncology.

## 1 Introduction

Cancer is the second leading cause of death worldwide, following cardiovascular diseases, which are responsible for millions of fatalities and the highest number of incidences each year. About 1 out of every 5 individuals developed cancer once in their lifetime. According to the World Health Organization (WHO), nearly 9.7 million cancer-related fatalities occurred in the year 2022. The cancer registry program ranked the cancer diseases based on incidence rates (Bray et al., 2024). Where in the list, lung cancer with 2.5 million new cases, breast cancer with 2.3 million new cases, colorectal cancer with 1.9 million new cases, prostate cancer with 1.5 million new cases, and stomach cancer with 970,000 new cases. In addition to that, the International Agency for Cancer Research (IACR) projected that approximately, 50 million new cancer-related cases will be predicted in the year 2050 (Sathishkumar et al., 2022).

Given this global burden and complexity of cancer, the identification of key oncogenic proteins and relevant biomarkers is crucial for understanding disease mechanisms, as well as on the development of new approaches, which is essential for the diagnosis and management of cancer. One such protein, polo-like kinase 1, or PLK1, is a well-characterized mitotic regulator, which belongs to the serine/threonine kinase family. It plays a key role in regulating various stages of cell cycle progression, which includes spindle formation, segregation of chromosomes, the G2/M checkpoint, mitotic entry controls, and DNA replication (Su et al., 2022). It has 603 amino acids (aa) and two key domains, which are the protein kinase domain (53-305 aa) and the polo-box domain (410-488 and 510-592 aa). The protein kinase domain (N-terminal) is responsible for phosphorylation, while polo box domains (C-terminal) is essential for substrate recognition and localization. Together, these domains maintain and regulate mitosis and cellular processes. Dysregulation acquired in these domains leads to genomic instability and epigenetic changes and inhibits the activity of tumor suppressors and apoptotic proteins, thus preventing degradation and persistent expression of PLK1 (Chiappa et al., 2022; Su et al., 2022; Lim et al., 2024). Previous studies have reported that the dysregulation of PLK1 is highly associated with a wide range of cancers, including breast, esophagus, stomach, lung, ovary, prostate, pancreas, head, and neck. Subsequently, the overexpression of PLK1 is frequently linked to poor prognosis and its predominantly limited treatment choices, making it a potential target for cancer diagnostics and therapeutics (Wierer et al., 2013; Kahl et al., 2022; Kandala et al., 2023; Wang et al., 2025).

Alterations in genes, particularly single nucleotide polymorphisms (SNPs), contribute to cancer diagnosis and management. These SNPs are often present in coding regions and highly elevate the levels of epigenetic profiles, which in turn potentially affect the protein functions. Compared to synonymous SNPs, non-synonymous SNPs (nsSNPs) have gained a lot of interest due to their capability in altering protein structure and function. These missense mutations might influence cancer progression, low survival outcomes, and poor prognosis (Navapour and Mogharrab, 2021; Khan et al., 2022). Recently, the PLK1 has emerged as a key oncogenic target in cancer research. Therefore, this study aims to identify possible and potential nsSNPs in the PLK1 using computational approaches and molecular dynamic simulations, which may aid in biomarker discovery and therapeutic intervention. Initially, the nsSNPs of PLK1 were retrieved for different cancer phenotypes utilizing a large set of cancer databases. Following that, a wide range of computational tools like SIFT, PolyPhen-2, E-SNPs and GO, MutPred2, FATHMM-XF, I-Mutant 2.0, CUPSAT, DynaMut2, mCSM, and so on were utilized for prioritizing high-risk nsSNPs of PLK1 in all types of cancers (Navapour and Mogharrab, 2021; Yadav and Singh, 2021; Kamal et al., 2024). Concurrently, the effects of mutation on protein structure were predicted by using molecular dynamic simulations (MDS). The structural stabilities of the complex were performed at the atomic level, by utilizing MDS, and its statistical measurements were calculated for both wild-type and mutant proteins, including root mean square deviations (RMSD), root mean square fluctuations (RMSF), solvent accessible surface area (SASA), and radius of gyration (ROG). Overall, this study concentrated on predicting deleterious and potential nonsynonymous single nucleotide polymorphisms (nsSNPs) in the PLK1 gene that alter protein functions and contribute to various types of cancer using *in silico* approaches.

## 2 Materials and methods

### 2.1 Kaplan-meier survival analysis of PLK1 expression in various types of cancer

Prior investigating the functional and structural impacts of nsSNPs in PLK1, it is import to determine whether the PLK1 expression itself is clinically relevant in human cancers. To address the prognostic relevance of PLK1 expression in different types of cancers, a Kapler-Meier (KM) survival analysis was conducted by using online web resources, including KMplotter (https://kmplot.com/analysis/index.php?p=home) and Gene Expression Profiling Interactive Analysis (GEPIA2) (http://gepia2.cancer-pku.cn/#index) (Tang et al., 2019; Győrffy, 2024). These tools contain clinical outcome data and gene expression data, which are deposited by large cohorts such as the Cancer Genome Atlas (TCGA) and Gene Expression Omnibus (GEO).

The gene, PLK1 (ENSG00000166851.14), was queried, and the overall survival (OS) outcomes and disease-free survival (DFS) were analyzed for bladder, breast, cervical, esophagus, head and neck, kidney, liver, lung, ovarian, pancreatic, rectum, sarcoma, stomach, thyroid, and uterine-related cancers. Based on the median expression of PLK1, the patients were stratified into low and high expression groups. The OS and DFS plots were reported along with log-rank probability values and the hazard ratio (HR) with 95% confidence intervals. A p-value less than 0.05 was considered statistically significant. By analyzing the survival outcomes, the correlation between PLK1 overexpression and poor patient prognosis was determined. This provides a strong rationale for investigation into PLK1’s genetic variants (nsSNPs), structural alterations and therapeutic targeting in cancer treatment.

### 2.2 Data collection

**FIGURE 1:**
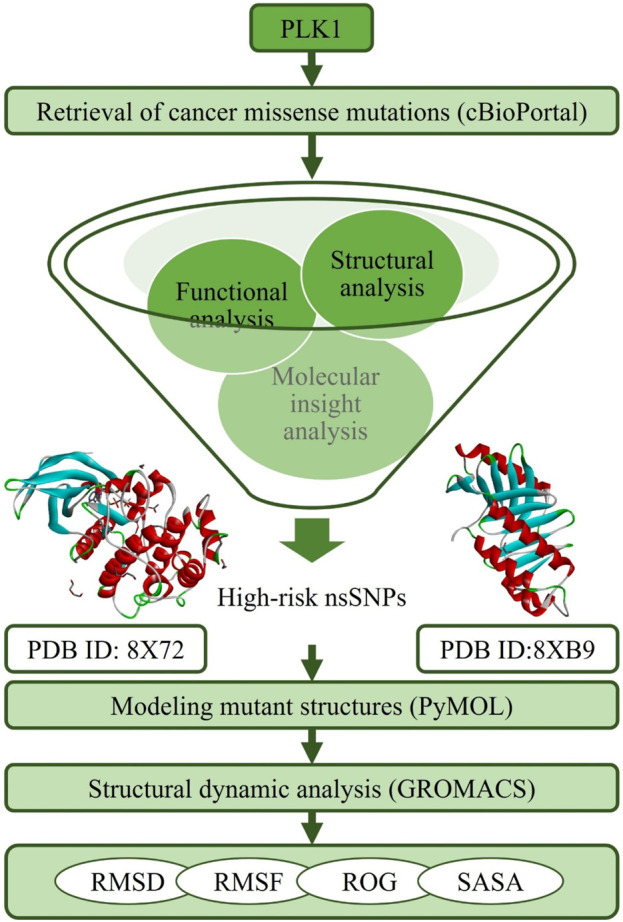
Schematic representation of the methodology used in screening oncogenic missense variants in PLK1.

The non-synonymous single nucleotide polymorphisms (nsSNPs) for the human PLK1 gene were acquired from cBioPortal (cBio Cancer genomic portal) for cancer genomics (https://www.cbioportal.org/), which contains a large set of human cancer clinical data deposited from TCGA PanCancer Atlas studies and a curated set of non-redundant studies (Gao et al., 2013). In this research, SNPs of PLK1 related to colorectal adenocarcinoma, breast, esophagus, stomach, skin, lung, kidney, head and neck, lung, brain, bladder, ovarian, fallopian tube, cervical, pancreatic, and prostate cancers were retrieved from cBioPortal. A total of 207 nsSNPs were subjected to further analysis after removing redundancy from 594 PLK1 SNPs. The details of the mutations information’s such as repository, cancer type, mutant type, variant allele frequency and mutation samples provide in Supplementary Table S1. The PLK1 sequence was retrieved from the UniProt database with an ID of P53350 (603 aa) (https://www.uniprot.org/uniprotkb/P53350/entry), while the protein 3D structures were retrieved from the RCSB PDB database with IDs of 8X72 (13-345 aa, 2.20 Å) (https://www.rcsb.org/structure/8X72) and 8XB9 (371-603 aa, 1.95 Å) (https://www.rcsb.org/structure/8XB9). The overall strategy followed in this research is given in Figure 1.

### 2.3 Functional analysis of nsSNPs

The functional effects of nsSNPs on the PLK1 were predicted through standard bioinformatic tools and web servers. The identification of functional consequences of nsSNPs is a pivotal step for understanding cancer disease mechanisms and developing targeted therapies. The computational tools utilized for prioritizing high-risk nsSNPs were Sorting Intolerant from Tolerant (SIFT) (https://sift.bii.a-star.edu.sg/), which categorizes the nsSNPs into deleterious or tolerant, and Polymorphism Phenotyping 2 (PolyPhen-2) (http://genetics.bwh.harvard.edu/pph2/), which predicts whether an nsSNP is damaging or benign (Ng, 2003; Adzhubei et al., 2010). The cancer-driven and pathogenic nsSNPs were predicted using Protein Variant (ProtVar) (https://www.ebi.ac.uk/ProtVar/), Computational prediction of the pathogenic status of cancer-specific somatic variants (CScape) (http://cscape.biocompute.org.uk/), Functional Analysis through Hidden Markov Models - eXtended Features (FATHMM-XF) (https://fathmm.biocompute.org.uk/fathmm-xf/), Embedding SNPs and Gene Ontology (E-SNPs&GO) (https://esnpsandgo.biocomp.unibo.it/), and Mutation Prediction (MutPred2) (http://mutpred2.mutdb.org/index.html) tools (Rogers et al., 2017; Rogers et al., 2018; Pejaver et al., 2020; Manfredi et al., 2022; Stephenson et al., 2024). All of these tools identify high-risk-associated nsSNPs of the PLK1 using various algorithms. Table 1 contains the information’s of missense SNPs functional predictors and description. By integrating these methods, potential and pathogenic nsSNPs were predicted. The variants that were consistently identified as highly deleterious or damaging and demonstrated pathogenic potential in most of the tools were subjected to further analysis.

**TABLE 1:** Missense SNPs functional predictors and description.

Tool name (acronym)	Description	Accessible link
Sorting Intolerant from Tolerant (SIFT)	It uses amino acid evolutionary conservations across related homologous sequences and predicts whether mutations are tolerated or deleterious	https://sift.bii.a-star.edu.sg/
Polymorphism Phenotyping 2 (PolyPhen-2)	It uses both sequence conservation and structural features such as solvent accessibility and secondary structure to classify if the mutation belongs to a damaging or benign class	http://genetics.bwh.harvard.edu/pph2/
Protein Variant (ProtVar)	This tool has information on protein sequence, structure, and functional annotation data. It evaluates the functional and clinical impacts of each amino acid substitution upon submission	https://www.ebi.ac.uk/ProtVar/
Computational prediction of the pathogenic status of cancer-specific somatic variants (CScape)	This tool uses a machine learning model, which is trained on a large dataset of cancer-related mutations, and predicts the potential pathogenic somatic variants in various types of cancers	http://cscape.biocompute.org.uk/
Functional Analysis through Hidden Markov Models - eXtended Features (FATHMM-XF)	It predicts pathogenicity of coding and non-coding variants in cancer using hidden Markov models with extended sequence or functional features	https://fathmm.biocompute.org.uk/fathmm-xf/
Embedding SNPs and Gene Ontology (E-SNPs&GO)	It predicts the functional impact of a mutation by integrating information of protein sequence with Gene Ontology (GO) terms	https://esnpsandgo.biocomp.unibo.it/
Mutation Prediction (MutPred2)	It’s a comprehensive tool that uses a machine learning model to forecast whether the mutations belong to a pathogenic or benign class and also provides the molecular mechanism of mutations that affect a binding site, catalytic activity, or a post-translational modification site	http://mutpred2.mutdb.org/index.html

### 2.4 Structural impact prediction of nsSNPs

To forecast the structural effects of nsSNPs on the PLK1, *in silico* tools and web servers were used. The list of tools that predict PLK1 stability changes upon amino acid substitutions includes I-Mutant 2.0 (https://folding.biofold.org/i-mutant/i-mutant2.0.html), Cologne University Protein Stability Analysis Tool (CUPSAT) (https://cupsat.brenda-enzymes.org/), Dynamically-informed Mutation Analysis (DynaMut2) (https://biosig.lab.uq.edu.au/dynamut2/), Mutation Cutoff Scanning Matrix (mCSM) (https://biosig.lab.uq.edu.au/mcsm/), Site-Directed Mutagenesis (SDM) (https://www-cryst.bioc.cam.ac.uk/∼sdm/sdm.php), and DUET (https://biosig.lab.uq.edu.au/duet/) (Capriotti et al., 2005; Parthiban et al., 2006; Worth et al., 2011; Pires et al., 2014b; 2014a; Rodrigues et al., 2021). These tools predict protein stability by calculating Gibbs free energy based on the difference between native and mutant protein structures. Among them, CUPSAT provides details on torsion angles, which are used for assessing the amino acid environment in the mutation site. On the other hand, the mCSM tool provides information on relative surface accessibility (RSA), which is crucial for assessing residue exposure to solvents. This exposure, in turn, influences the impact of mutations on protein stability and binding affinity. Table 2 contains the information’s of missense SNPs structural predictors and description. The use of these tools allows for the prediction and prioritization of nsSNPs that impact PLK1 stability with high accuracy. Furthermore, the mutants identified as highly destabilizing in the majority of tools were utilized for further investigations.

**TABLE 2:** Missense SNPs structural predictors and description.

Tool name (acronym)	Description	Accessible link
I-Mutant 2.0	It predicts stability changes upon amino acid substitutions using support vector machines trained on the ProTherm thermodynamic database	https://folding.biofold.org/i-mutant/i-mutant2.0.html
Cologne University Protein Stability Analysis Tool (CUPSAT)	It predicts stability changes for amino acid substitutions similar to I-Mutant but provides detailed information on torsion angles and local backbone effects	https://cupsat.brenda-enzymes.org/
Dynamically-informed Mutation Analysis (DynaMut2)	This tool uses normal mode analysis and graph-based signatures to evaluate mutation effects on protein flexibility and dynamics and also predicts the change in vibrational entropy, which indicates the rigidity or flexibility of a protein	https://biosig.lab.uq.edu.au/dynamut2/
Mutation Cutoff Scanning Matrix (mCSM)	It uses graph-based structural signatures of atomic distance patterns to predict stability and relative surface accessibility (RSA) changes for each amino acid substitution	https://biosig.lab.uq.edu.au/mcsm/
Site-Directed Mutagenesis (SDM)	This tool uses statistical potentials, which are derived from environment-specific substitution tables, and predicts the protein stability changes upon mutation	https://www-cryst.bioc.cam.ac.uk/∼sdm/sdm.php
DUET	It’s a comprehensive tool that combines information from mCSM and SDM. It improves the accuracy of protein stability predictions through these tools	https://biosig.lab.uq.edu.au/duet/

### 2.5 Prediction of molecular mechanisms and evolutionary conservation profiles

The molecular mechanisms of the screened nsSNPs were predicted by the MutPred 2 tool. It provides the information on affected PROSITE and ELM motifs along with molecular mechanisms with probability value set to be ≤0.05. The predicted molecular mechanism with a *p*-value less than 0.05 was signified as high confidence, while the value less than 0.01 was signified as confident alterations that impact protein function and structure upon amino acid substitutions. The evolutionarily conserved regions of amino acid substitutions were identified using the conservation surface-mapping (ConSurf) server (https://consurf.tau.ac.il/consurf_index.php), which is an open-source web server (Ashkenazy et al., 2016). Initially, the protein sequence was queried to distinguish extremely variable and highly conserved regions, with scores ranging from 1 to 9 based on a Bayesian algorithm (Yadav and Singh, 2021).

### 2.6 Modeling and structural evaluation of mutant PLK1 proteins

**FIGURE 2:**
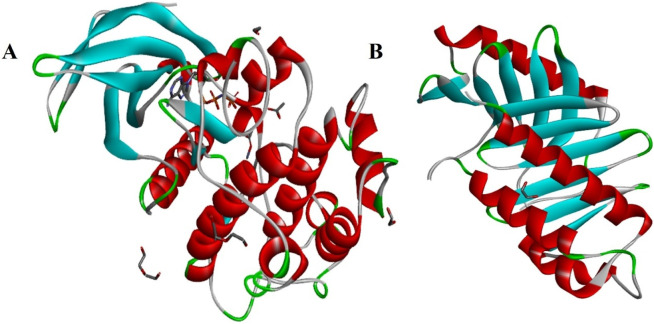
3D structures of PLK1 protein, where **(A)** Kinase domain containing PLK1 protein (PDB ID:8X72), **(B)** Polo-box domain containing PLK1 protein (PDB ID: 8XB9).

The screened nsSNPs were modeled through functional and structural analysis using open-source PyMol 3.1 software (https://www.pymol.org/). The wizard presented in this software is used to generate mutant protein structures. The mutant PLK1 were modeled using RCSB PDB IDs of 8X72 and 8XB9, and the structures were saved in a protein data format. The 3D structures are shown in Figure 2. Following that, the root mean square deviation for each mutant PLK1 and wild-type PLK1 was calculated using the TM-align tool, which analyzes residue-to-residue alignment based on the similarity of both the mutant and the wild-type PLK1 using a heuristic dynamic program (Zhang, 2005).

The structural consequences of the nsSNPs on the PLK1 were analyzed through the Have (y)our Protein Explained (HOPE) web server (https://www3.cmbi.umcn.nl/hope/) (Venselaar et al., 2010). This application examined the origin of diseases at the molecular level, which is related to the phenotype that is caused by mutant proteins. For each query amino acid substitution, it generates structural information by integrating data from 3D protein structure, annotations of sequences from the UniProt database, and predictions from Reprof software.

### 2.7 Analysis of relative surface accessibility and secondary structure elements in PLK1 mutants

The secondary structure, relative surface accessibility (RSA), disorder, and dihedral angles of each amino acid residue in the PLK1 were analyzed using NetSurfP 3.0 (https://services.healthtech.dtu.dk/services/NetSurfP-3.0/) (Høie et al., 2022; Kamal et al., 2024). This open-source software utilizes machine learning and natural language processing techniques. Understanding the secondary structure and RSA provides information on mutations, which may be tolerated if located on the surface or could disrupt protein folding and interactions if buried. Through this information, disease-causing nsSNPs were identified by examining whether the residues are located in the protein core or exposed on the surface.

### 2.8 Analysis of protein-protein network and effects of mutations on PPI interfaces

The STRING (Search Tool for the Retrieval of Interacting Genes/Proteins) database (https://string-db.org/) was used to examine the interactions between PLK1 and associated proteins along with information on gene fusion, co-occurrence, co-expression, experimental data, and biochemical interactions and determine a confidence score, which ranges from 0 (very low confidence) to 1 (very high confidence) (Szklarczyk et al., 2023). Protein-protein interactions (PPI) also forecast the query protein’s functional enrichments, which include protein domains and features, subcellular localization, tissue expression, KEGG pathways, Reactome pathways, Wiki pathways, and gene ontology (biological, cellular, and molecular processes).

To evaluate the identified variants and alter PPI interactions, the SAAMBE-3D server was utilized for evaluating PLK1 variants (Pahari et al., 2020). It estimates changes in binding free energy upon introducing point mutations. The mutations with positive DDG values were determined to be of a destabilizing nature, whereas the negative values indicated stabilizing effects. Following that, the protein-protein docking between PLK1 and interacting partners was observed using the ClusPro 2.0 server, which predominately applies rigid-body docking, clusters low-energy complexes, and refines them by energy minimization to obtain the most representative docked models (https://cluspro.org/login.php?redir=/home.php) (Jones et al., 2022). Then, the complexes were visualized using the PDBsum server, which provides a graphical summary of protein-protein interfaces such as hydrogen bonds, salt bridges, and interacting residues (https://www.ebi.ac.uk/thornton-srv/software/PDBsum1/) (Laskowski, 2022). This visualization validates whether the mutations affected the binding regions with interacting proteins.

### 2.9 Analysis of post translational modifications

Further, the potential alterations in post-translational modifications (PTM) present in wild-type and mutant variants were analyzed using PhosphoSitePlus (PSP), a freely available database that contains experimentally determined PTM in human and mouse proteins, and the MusiteDeep tool, a deep learning-based predictor of multiple PTM types, which includes phosphorylation, ubiquitination, acetylation, methylation, and glycosylation (Hornbeck et al., 2015; Wang et al., 2021). Predictions with scores above the threshold values were considered as potential PTM sites.

### 2.10 Structural stability analysis using molecular dynamic simulations

The stability of mutant PLK1 and native PLK1 structures was evaluated using molecular dynamics simulations (MDS). To investigate the structural effects on both mutant PLK1 and native PLK1, this study employed GROMACS software (Lemkul, 2019). The structural effects of mutations on the PLK1 protein were analyzed using the CHARMM27 all-atom force field, followed by generating the topology, adding a cubic box, incorporating solvent, adding appropriate ions, and performing energy minimization and equilibration steps. Initially, TIP3P water was added to solvate the system, and then the system was neutralized using counterions such as 0.15 M NaCl. Following that, the first equilibration was performed under NVT (number of particles, volume, temperature) at 300K, and the second equilibration step was performed under NPT (number of particles, pressure, temperature) with Parrinello-Rahman pressure coupling at 1 bar. A time step of 2 femtoseconds (fs) was used for all simulations. The final MDS production was conducted for 100 ns, with trajectories recorded for every 10 ps. The final MDS production was conducted for 100 ns, with trajectories recorded for every 10 ps. (Sinha and Wang, 2020; Abdalla et al., 2022; Sinha et al., 2022b; Subramani and Venugopal, 2024). Through MDS, a list of parameters was calculated and visualized to analyze the significant difference between mutant and wild-type PLK1 proteins, which includes root mean square deviation (RMSD), root mean square fluctuations (RMSF), solvent accessibility surface area, radius (SASA) of gyration (ROG) and hydrogen bond (H-bond). Further, these performances were visually interpreted using XMgrace software. Understanding the structural consequences of these mutations is crucial for assessing their impact on the PLK1. This information is further leveraged to develop targeted treatment strategies for candidates.

## 3 Results

### 3.1 Survival analysis of PLK1 expression

To establish the clinical relevance of PLK1, its expression was first evaluated as a prognostic marker in different types of cancer utilizing KM survival plots (number of patients = 7,489). The high level of PLK1 expression associated with poorer overall survival (OS) outcome for cancer patients, whereas low expression indicates the OS is not significantly correlated, further suggesting that the cancer types are not at high risk (Alzahrani et al., 2020). The most significant cancer types were observed to be kidney renal papillary carcinoma (HR = 6.69, p = 3.1e-11), kidney renal clear cell carcinoma (HR = 2.92, p = 2.1e-13), pancreatic ductal adenocarcinoma (HR = 2.0, p = 0.00077), lung adenocarcinoma (HR = 2.07, p = 7.8e-06), breast (HR = 1.41, p = 2.9e-11), uterine corpus endometrial carcinoma (HR = 1.99, p = 0.005), sarcoma (HR = 2.09, p = 0.0047), head and neck (HR = 1.32, p = 0.042), and bladder (HR = 1.43, p = 0.016) cancers. Subsequently, high expression of PLK1 correlated with improved OS in lung squamous cell carcinoma (HR = 0.71, p = 0.013), while no significant correlation was observed between OS and PLK1 expression in rectal, stomach, esophageal squamous, and ovarian cancers. Details about overall survival analysis for all type cancers are shown in Supplementary Figure S1. Furthermore, no statistically significant correlation between PLK1 expression and OS was observed in thyroid, esophageal adenocarcinoma, and cervical cancers.

**FIGURE 3:**
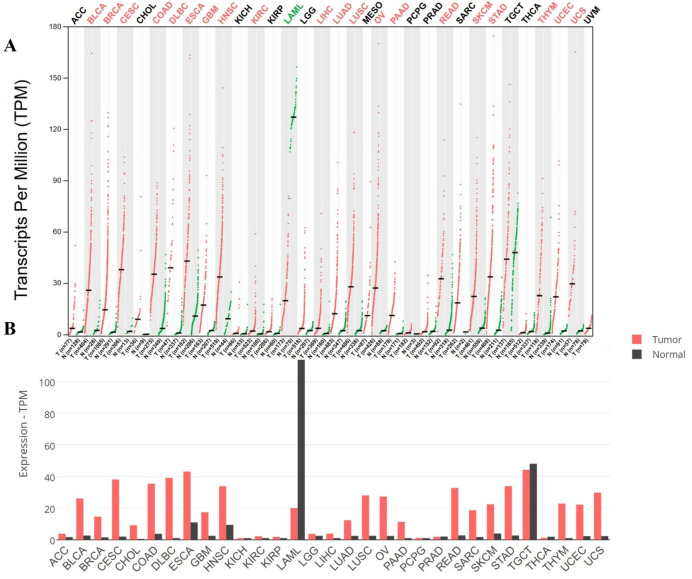
Expression profiles of PLK1 across cancer types predicted through GEPIA2, where in the **(A)** Dot plot, red dots represent tumor tissue expression, green dots represent paired normal tissue expression from the same patients, and black bars represent unpaired normal tissue expression from healthy controls, and in the **(B)** Box plot, red and black represent tumor and normal tissues.

**FIGURE 4:**
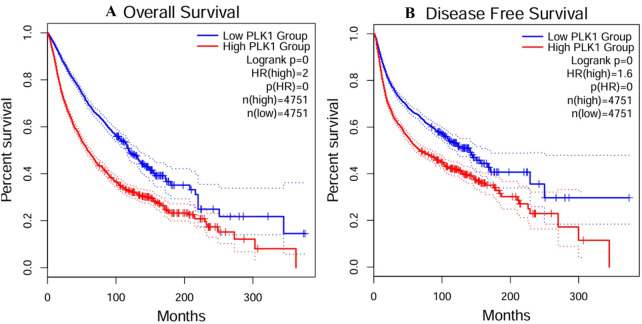
Survival analysis of PLK1 expression across all type of cancers predicted through GEPIA2, where **(A)** Overall survival outcomes, and **(B)** Disease free survival.

The GEPIA2 demonstrated that PLK1 was significantly overexpressed in a variety of tumors compared to normal tissues. The dot plot displayed elevated levels of PLK1 observed in breast (BRCA), liver (LIHC), and lung adenocarcinoma (LUAD) as indicated by transcript per millions (TPM). The box plot highlights the consistent increase in esophageal carcinoma and testicular germ cell tumors, followed by moderate expression in rectum, cervical, lung, and breast tissues compared to normal tissues. Figure 3 showcases visual representation of PLK1 expression in tumor and normal tissues. Further, the OS and DFS were determined for all types of cancers and the OS and DFS data are shown in Figure 4. For OS, patients with high PLK1 expression (n = 4,751) exhibited significantly poorer survival outcomes (HR = 2.0, p-value <0.0001), while DFS showed that high levels of PLK1 expression were associated with reduced survival (HR = 1.6, p-value <0.0001). Together, these findings demonstrated that the overexpression of PLK1 is highly associated with poor survival outcomes in multiple cancers, which validates its role as a prognostic biomarker. More importantly, these results provide clinical rationale for investigating nsSNPs, structural alterations, and potential therapeutic targeting of PLK1, which were addressed in the following sections.

### 3.2 Functional prediction of high-risk nsSNPs

The SNPs of PLK1 were retrieved from 438 different cancer studies by querying 147,306 samples. Afterwards, 207 cancer-associated missense SNPs from cBioPortal were analyzed by the SIFT server, which predicted 104 variants that had a potential impact on PLK1 function and provides normalized probability scores ranging from 0 to 1. The deleterious prediction of missense variants is shown in Supplementary Table S2. The predicted score near zero indicates deleterious nsSNPs, while the score near 1 indicates tolerated nsSNPs, which do not impact the PLK1 function. Following that, PolyPhen-2 predicted that 90 nsSNPs were probably damaging to the function and structure of the PLK1 protein, with a probability score near 1. The damaging missense variants predictions are shown in Supplementary Table S3. It calculates the score and predicted the functional significance of the allele using the Naïve Bayes algorithm with a trained dataset containing HumDiv and HumVar discrepancies (Dhakar et al., 2022; Khan et al., 2022).

To screen the pathogenic nsSNPs, ProtVar, CScape, and FATHMM-XF tools were utilized upon querying chromosome numbers, coordinates, reference, and alternative alleles of screened nsSNPs on the submission page. ProtVar from EMBL-EBI of the European Bioinformatics Institute predicted the variants that impact protein function and thus affect human health. This tool contains integrated genomic and proteomic prediction results, which were retrieved from CADD v.1.7 and AlphaMissense tools. The CADD score above 20 and AlphaMissense score above 0.56 were chosen for further screening, as they indicate potentially deleterious and pathogenic variants (Stephenson et al., 2024). CScape predicted oncogenic and benign variants using a random forest classifier algorithm by generating features from the COSMIC and dbSNP databases, which were used for analyzing both coding and non-coding regions of the entire genome. On the other hand, FATHMM-XF predicted variants presented in coding regions and classified them into pathogenic and benign. The prediction results are shown in Supplementary Table S4. A total of 71 variants with a probability value exceeding 0.5 were identified as potential pathogenic and oncogenic nsSNPs. Subsequently, the variants were further screened using MutPred 2 and E-SNPs and GO tools by querying amino acid substitutions. Table 3 provides the pathogenic predictions score for mutations using E-SNPs and GO and MutPred2 tools. As a result, pathogenic (*p* score >0.5) and benign (*p* score <0.5) variants were predicted. Furthermore, the E-SNPs and GO tool acquired information on the reliability index (RI), which indicates the confidence level of the prediction. This index generates a numerical score ranging from 0 to 10, where an RI score above 0.5 suggests disease-associated nsSNPs, and a higher RI indicates a more reliable prediction (Dash et al., 2020). Through this analysis, 51 nsSNPs were identified as highly pathogenic, which could impact PLK1 functions.

**TABLE 3:** Pathogenicity predictions of missense variants in PLK1 using E-SNPs & GO and MutPred2 tools.

S. No	Amino acid change	E-SNPs and GO			MutPred 2	
		Pathogenicity	Pathogenicity probability (pathogenic: ≥0.5)	Reliability index (RI)	Prediction	Score
1	P41Q	Benign	0.38	2	Pathogenic	0.628
2	E101D	Pathogenic	0.93	9	Benign	0.449
3	H110R	Pathogenic	0.87	7	Pathogenic	0.821
4	E121G	Pathogenic	0.82	6	Pathogenic	0.891
5	E152K	Pathogenic	0.93	9	Pathogenic	0.908
6	R154L	Pathogenic	0.82	6	Pathogenic	0.911
7	R154Q	Pathogenic	0.71	4	Pathogenic	0.816
8	C164Y	Pathogenic	0.48	0	Pathogenic	0.687
9	R175G	Pathogenic	0.85	7	Pathogenic	0.93
10	R175P	Pathogenic	0.87	7	Pathogenic	0.956
11	R175Q	Pathogenic	0.86	7	Pathogenic	0.864
12	L188P	Pathogenic	0.47	1	Pathogenic	0.944
13	V190M	Benign	0.27	5	Pathogenic	0.866
14	G196E	Pathogenic	0.93	9	Pathogenic	0.946
15	G196V	Pathogenic	0.93	9	Pathogenic	0.958
16	T199I	Benign	0.41	2	Pathogenic	0.92
17	K208E	Pathogenic	0.78	6	Pathogenic	0.867
18	G213V	Pathogenic	0.92	8	Pathogenic	0.93
19	P220A	Pathogenic	0.84	7	Pathogenic	0.818
20	P220L	Pathogenic	0.89	8	Pathogenic	0.894
21	E231K	Pathogenic	0.72	4	Pathogenic	0.865
22	D233G	Pathogenic	0.93	9	Pathogenic	0.947
23	D233Y	Pathogenic	0.93	9	Pathogenic	0.948
24	W235L	Pathogenic	0.84	7	Pathogenic	0.941
25	L244F	Pathogenic	0.56	1	Pathogenic	0.854
26	E252Q	Benign	0.37	3	Pathogenic	0.626
27	E258Q	Benign	0.17	7	Benign	0.467
28	R262W	Benign	0.46	1	Pathogenic	0.687
29	I263M	Benign	0.41	2	Pathogenic	0.754
30	A278D	Pathogenic	0.56	1	Pathogenic	0.889
31	R293C	Pathogenic	0.93	9	Pathogenic	0.883
32	R293H	Pathogenic	0.93	9	Pathogenic	0.841
33	F304L	Pathogenic	0.78	6	Pathogenic	0.637
34	F304V	Pathogenic	0.84	7	Pathogenic	0.684
35	P311L	Pathogenic	0.93	9	Pathogenic	0.619
36	L319M	Benign	0.13	7	Benign	0.291
37	P323L	Benign	0.36	3	Pathogenic	0.808
38	R337W	Pathogenic	0.62	2	Benign	0.387
39	P339L	Benign	0.24	5	Pathogenic	0.57
40	P407L	Pathogenic	0.82	6	Pathogenic	0.753
41	D416Y	Pathogenic	0.96	9	Pathogenic	0.9
42	S418L	Pathogenic	0.95	9	Pathogenic	0.821
43	Y421N	Pathogenic	0.89	8	Pathogenic	0.924
44	G422R	Pathogenic	0.95	9	Pathogenic	0.918
45	Y425H	Pathogenic	0.95	9	Pathogenic	0.872
46	Q426P	Pathogenic	0.98	10	Pathogenic	0.948
47	D429H	Pathogenic	0.62	2	Pathogenic	0.888
48	V432M	Benign	0.46	1	Pathogenic	0.695
49	G433E	Pathogenic	0.98	10	Pathogenic	0.935
50	Y485H	Pathogenic	0.94	9	Pathogenic	0.858
51	M486I	Pathogenic	0.46	1	Pathogenic	0.859
52	H489Q	Pathogenic	0.54	1	Pathogenic	0.7
53	L490W	Pathogenic	0.86	7	Pathogenic	0.765
54	R507W	Benign	0.36	3	Pathogenic	0.718
55	R516C	Pathogenic	0.84	7	Pathogenic	0.753
56	T517I	Pathogenic	0.62	2	Pathogenic	0.714
57	A520T	Pathogenic	0.62	2	Pathogenic	0.861
58	L525F	Benign	0.38	2	Pathogenic	0.892
59	L525P	Pathogenic	0.96	9	Pathogenic	0.967
60	Q531H	Pathogenic	0.93	9	Pathogenic	0.902
61	N533K	Pathogenic	0.93	9	Pathogenic	0.896
62	N533S	Benign	0.46	1	Pathogenic	0.75
63	F534I	Pathogenic	0.8	6	Pathogenic	0.823
64	V550M	Benign	0.26	5	Pathogenic	0.829
65	R557Q	Benign	0.13	7	Pathogenic	0.675
66	R557W	Pathogenic	0.51	0	Pathogenic	0.756
67	R563C	Benign	0.24	5	Benign	0.416
68	R579Q	Pathogenic	0.86	7	Pathogenic	0.697
69	R579W	Pathogenic	0.95	9	Pathogenic	0.782
70	Y582C	Pathogenic	0.87	7	Pathogenic	0.797
71	A583T	Benign	0.36	3	Pathogenic	0.591

### 3.3 Structural impact assessment of high-risk nsSNPs

**FIGURE 5:**
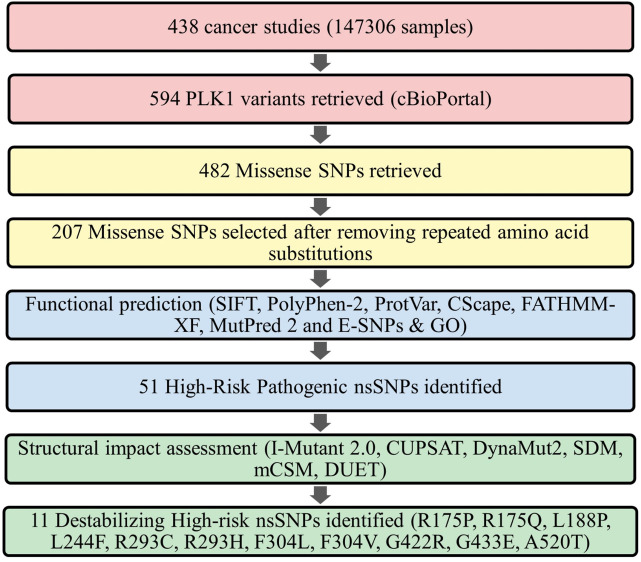
Flowchart of screening missense variants in PLK1.

**FIGURE 6:**
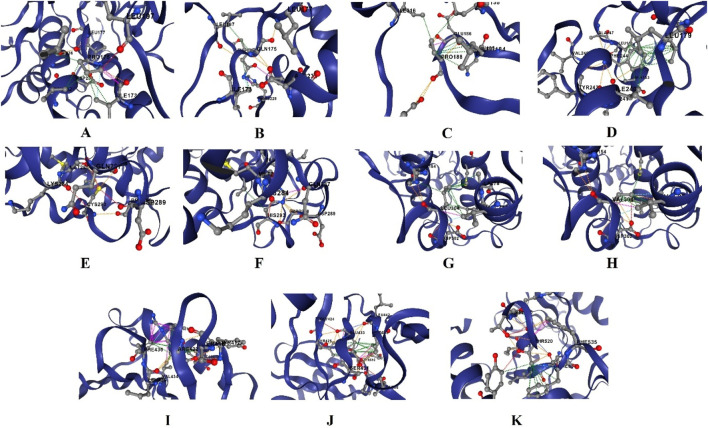
Close up view of PLK1 mutation structures viewed through DynaMut2, where **(A)** R175P, **(B)** R175Q, **(C)** L188P, **(D)** L244F, **(E)** R293C, **(F)** R293H, **(G)** F304L, **(H)** F304V, **(I)** G422R, **(J)** G433E, and **(K)** A520T.

The functional screened high-risk-associated nsSNPs were assessed for their role in the structural impact of PLK1. I-Mutant 2.0, CUPSAT, and DynaMut2, mCSM, SDM, and DUET tools were employed for screening the nsSNPs that highly destabilize the PLK1 structure. The structural predictions of missense variants are shown in Table 4. These tools determine the protein stability upon providing changes in Gibbs free energy (DDG) between wild-type and mutant PLK1 proteins. The DDG (kcal/mol) with a negative score indicates a decrease in stability and an unstable interaction in the PLK1 structure, while a positive score suggests that the single point mutation does not impact any structural changes in the PLK1. The I-Mutant and CUPSAT predicted DDG upon mutations, where the negative values strongly indicate destabilization; for instance, L188P and R293C were predicted to strongly destabilize the protein structure. The DynaMut2 incorporates normal mode analysis along with assessing the torsion flexibility and structural destabilization, which was observed in critical residues of R175 variants and L244F. On the other hand, mCSM used graph-based signatures to assess the RSA, suggesting whether the mutations occurred at exposed or buried regions; for example, the predicted highly destabilizing mutations were W235L, L525P, and R175G. Concurrently, the DUET tool consistently predicted R175, R293, G422, G433, and L525 variants as highly destabilizing with DDG scores greater than −2.0. Through these analyses, 11 missense SNPs were predicted as highly destabilizing for the PLK1 structure as well as greatly affecting the PLK1 protein’s nature. The screened list of high-risk-associated and cancer-causing nsSNPs in the PLK1 includes R175P, R175Q, L188P, L244F, R293C, R293H, F304L, F304V, G422R, G433E, and A520T. The overall flowchart of the missense mutations screening is showcases in Figure 5. For each mutant PLK1 structure, the close-up views are shown in Figure 6, which was visualized using the DynaMut2 server.

**TABLE 4:** Structural predictions of missense variants in PLK1.

S.No	Amino acid change	I-mutant 2.0		CUPSAT			DynaMut 2		mCSM			SDM		DUET	
		DDG (Kcal/mol)	Stability	DDG (Kcal/mol)	Stability	Torsion	DDG (Kcal/mol)	Stability	RSA (%)	DDG (Kcal/mol)	Stability	DDG (Kcal/mol)	Stability	DDG (Kcal/mol)	Stability
1	H110R	0.07	Increase	−2.1	DS	F	−0.2	DS	32.7	−0.8	DS	−1	DS	−1	DS
2	E121G	−1.48	Decrease	−3.1	DS	UF	−0.7	DS	52	−0.9	DS	0.6	S	−1	DS
3	E152K	−0.88	Decrease	−1.3	DS	F	0.7	S	0	0.85	S	−2	DS	0.8	S
4	R154L	−0.01	Decrease	3.37	S	F	−0.9	DS	6	−1.3	DS	0.8	S	−1	DS
5	R154Q	−0.54	Decrease	1.52	S	F	−0.9	DS	6	−1.4	DS	−2	DS	−2	DS
6	C164Y	0.16	Increase	−0.3	DS	UF	−1.1	DS	0	−1.3	DS	−1	DS	−1	DS
7	R175G	−0.68	Decrease	0.38	S	F	−1.6	DS	8.9	−2.1	Highly DS	−1	DS	−2	DS
8	R175P	−0.62	Decrease	−3.5	DS	UF	−0.6	DS	8.9	−0.9	DS	−3	DS	−2	DS
9	R175Q	−0.58	Decrease	−6.3	DS	UF	−0.9	DS	8.9	−1.4	DS	−2	DS	−2	DS
10	L188P	−2.42	Decrease	−2.1	DS	UF	−2	DS	0.4	−1.8	DS	−3	DS	−2	DS
11	G196E	−0.67	Decrease	1.33	S	F	−1.3	DS	35.5	−1.4	DS	0.3	S	−1	DS
12	G196V	−2.05	Decrease	−1.5	DS	UF	−1.5	DS	35.5	−0.5	DS	0.5	S	−0	DS
13	K208E	−0.3	Decrease	0.13	S	UF	−0.7	DS	16.1	−0.7	DS	−1	DS	−1	DS
14	G213V	−1.63	Decrease	−2.9	DS	UF	−1.6	DS	66.2	−0.4	DS	0.7	S	0.1	S
15	P220A	−2.41	Decrease	3.78	S	F	−1.2	DS	3.4	−1.6	DS	2.6	S	−1	DS
16	P220L	−1.92	Decrease	6.29	S	F	−0.2	DS	3.4	−0.1	DS	1.8	S	0.6	S
17	E231K	−1.48	Decrease	−1.7	DS	F	−1	DS	29.6	−1.5	DS	−1	DS	−1	DS
18	D233G	−2.08	Decrease	1.44	S	UF	−1	DS	0	−1.6	DS	−1	DS	−2	DS
19	D233Y	−0.39	Decrease	1.81	S	F	−1.1	DS	0	−1	DS	−1	DS	−1	DS
20	W235L	−1.13	Decrease	2.35	S	F	−3.1	DS	0.2	−3	Highly DS	−3	DS	−3	DS
21	L244F	−0.32	Decrease	−1.3	DS	UF	−1.7	DS	0.9	−1.7	DS	−1	DS	−2	DS
22	A278D	0.02	Increase	−3.6	DS	UF	−1.7	DS	0.3	−1.8	DS	−3	DS	−2	DS
23	R293C	−0.51	Decrease	−12	DS	UF	−1.4	DS	0.1	−1.9	DS	−3	DS	−2	DS
24	R293H	−0.86	Decrease	−2.5	DS	UF	−1.3	DS	0.1	−1.9	DS	−2	DS	−2	DS
25	F304L	−2.08	Decrease	−2.7	DS	UF	−1.8	DS	1.4	−1.1	DS	−2	DS	−1	DS
26	F304V	−2.89	Decrease	−2.4	DS	UF	−1.8	DS	1.4	−1.7	DS	−3	DS	−2	DS
27	P311L	−1.12	Decrease	−0.5	DS	UF	−0.5	DS	2	−0.3	DS	0.4	S	0.2	S
28	P407L	0.48	Increase	−3.5	DS	UF	−0.5	DS	2.4	−0.8	DS	−0	DS	−0	DS
29	D416Y	−0.79	Decrease	0.84	S	UF	2.6	S	17.2	2.22	Highly S	0.5	S	1.9	S
30	S418L	−1.21	Decrease	−1	DS	F	−0.7	DS	29.1	−0.3	DS	1.5	S	0.3	S
31	Y421N	−1.95	Decrease	1.22	S	UF	−1.5	DS	51.1	−1.8	DS	0	DS	−2	DS
32	G422R	−0.62	Decrease	−9.6	DS	UF	−0.8	DS	2.2	−0.8	DS	−3	DS	−1	DS
33	Y425H	−1.27	Decrease	−4.1	DS	F	−1.8	DS	1.5	−2	Highly DS	−1	DS	−2	DS
34	Q426P	−0.58	Decrease	1.5	S	UF	−0	DS	10.9	−0	DS	−1	DS	0	S
35	D429H	−0.87	Decrease	−1	DS	F	−0.1	DS	35.1	−0.7	DS	0	S	−0	DS
36	G433E	−0.39	Decrease	−0.1	DS	UF	−1.5	DS	0.2	−1.7	DS	−2	DS	−2	DS
37	Y485H	−1.12	Decrease	−0.1	DS	UF	−1.3	DS	33.6	−1.8	DS	0.1	S	−2	DS
38	M486I	0.32	Increase	−0.5	DS	F	−0.4	DS	1.3	−0.9	DS	−0	DS	−1	DS
39	H489Q	−0.6	Decrease	−0.7	DS	UF	0.1	S	53.8	−0.1	DS	0	S	0	S
40	L490W	−0.94	Decrease	0.17	S	F	−0.4	DS	34.9	−1.3	DS	−0	DS	−1	DS
41	R516C	−1.05	Decrease	−2.8	DS	F	−1.5	DS	42.5	−1.8	DS	−0	DS	−2	DS
42	T517I	−0.32	Decrease	−1.2	DS	UF	0.1	S	31.9	−0.1	DS	1.1	S	0.4	S
43	A520T	−0.76	Decrease	−3.8	DS	UF	−0.6	DS	0	−0.9	DS	−2	DS	−1	DS
44	L525P	−1.07	Decrease	−4.2	DS	F	−1.5	DS	0.9	−2	Highly DS	−4	DS	−3	DS
45	Q531H	−1.36	Decrease	0.14	S	UF	−1	DS	1	−1.4	DS	0.8	S	−1	DS
46	N533K	−1.91	Decrease	0.95	S	UF	−0.5	DS	6.7	−0.2	DS	−2	DS	−0	DS
47	F534I	−1.46	Decrease	−1	DS	F	−1.9	DS	8	−1.5	DS	0.1	S	−1	DS
48	R557W	−0.78	Decrease	−2.9	DS	UF	−0.6	DS	48.8	−0.3	DS	0.2	S	−0	DS
49	R579Q	−0.92	Decrease	0.75	S	F	−1.6	DS	18.2	−1.5	DS	−1	DS	−1	DS
50	R579W	−0.5	Decrease	1.58	S	UF	−1.2	DS	18.2	−0.9	DS	0.4	S	−1	DS
51	Y582C	0.65	Increase	−0.5	DS	UF	−0.2	DS	35.8	−1.3	DS	−0	DS	−1	DS

Where, DDG-Gibb’s free energy, S-Stabilizing, DS-Destabilizing, F-Favourable, UF-Unfavourable, RSA-Relative surface accessibility.

### 3.4 Evaluation of molecular mechanism and conservation profiles of mutant residues

**FIGURE 7:**
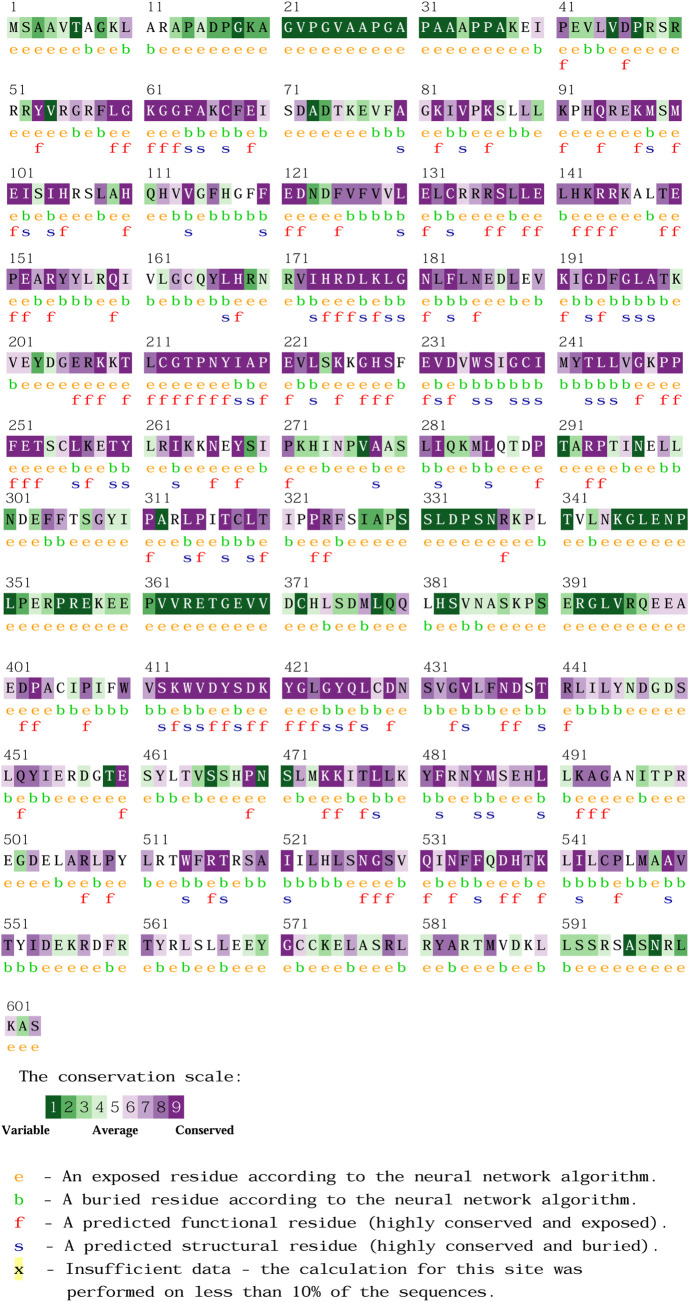
Conservation profile of PLK1 predicted through CornSurf server.

The molecular mechanism and overview of predicted PLK1 cancer mutations were analyzed by MutPred2. The predominant mechanisms include gain or loss of catalytic or allosteric sites, gain or loss of secondary structure, altered metal or DNA binding and others. It also forecasts the posterior probability of each amino acid substitution to evaluate the likelihood of altered molecular property, in terms of increased or decreased function. Further, the conservation profile for each amino acid residue was analyzed by the ConSurf server. The analysis of ConSurf is shown in Figure 7. Understanding the evolutionary information is crucial for determining whether the variants were highly cancer-associated mutations or not. Information on conserved (high or low) regions, exposed or buried residue types, and functional or structural residues for amino acid positions was determined. With these conservation profiles, the effects of amino acids on PLK1 protein structure and function were validated, thereby facilitating targeted treatment strategies. The mutations, such as R175P/Q, were found to be highly conserved, exposed, and functional sites, which were predicted to lose allosteric sites at D176, alter DNA/metal binding, and gain a catalytic site at K178. Similarly, the mutation R293 C/H also occurred at a highly conserved, exposed, and functional residue, which lost the allosteric site at the residue of R293. On the other hand, G4222R and G433E were located at highly conserved, exposed, and functional residues, which were predicted to induce gain of glycosylation at N437, loss of sulfation at Y417, and altered protein-protein interactions. In contrast, the variants such as L188P, L244F, and A520T were found in buried regions with moderate to high conservation, which highlights structural roles. They destabilize PLK1 upon altering ordered interfaces, secondary structural elements, or loop regions. On the other hand, the mutation F304 L/V did not cause any significant mechanistic disruptions, indicating that its effects on protein function and structure were neutral. The molecular mechanisms and conservation profiles of highly deleterious, pathogenic and structurally destabilizing missense variants are shown in Table 5.

**TABLE 5:** Molecular mechanisms and conservation profiles of highly deleterious, pathogenic and structurally destabilizing missense variants.

S No	Amino acid change	MutPred 2				CornSurf conservation profile
		Affected PROSITE and ELM motifs	Molecular mechanisms with P-values ≤0.05	Probability	*P-*value	
1	R175P	ELME000106, ELME000134, ELME000146, ELME000155, ELME000233, PS00108	Loss of Allosteric site at D176	0.36	1.10E-03	Highly conserved, exposed and functional residue
			Gain of Strand	0.28	0.01	
			Altered Metal binding	0.27	4.60E-03	
			Gain of Loop	0.27	0.03	
			Altered DNA binding	0.23	9.50E-03	
			Gain of Catalytic site at K178	0.14	0.03	
2	R175Q	ELME000106, ELME000134, ELME000146, ELME000233, PS00108	Loss of Allosteric site at D176	0.32	2.80E-03	Highly conserved, exposed and functional residue
			Altered Metal binding	0.3	4.30E-03	
			Gain of Strand	0.27	0.03	
			Altered DNA binding	0.23	0.01	
			Gain of Catalytic site at K178	0.19	0.01	
3	L188P	None	Gain of Strand	0.3	3.10E-03	Average conserved, and buried residue
			Altered Ordered interface	0.28	0.04	
			Gain of Relative solvent accessibility	0.26	0.03	
			Altered Metal binding	0.22	0.03	
			Gain of Allosteric site at K191	0.19	0.05	
4	L244F	ELME000020, ELME000052, ELME000120, ELME000155, ELME000182, ELME000335	Altered Ordered interface	0.24	0.04	Highly conserved, buried and structural residue
			Gain of Allosteric site at Y242	0.23	0.02	
			Altered Transmembrane protein	0.11	0.03	
			Altered Metal binding	0.04	0.05	
5	R293C	ELME000053, ELME000062, ELME000064, ELME000147, ELME000220, PS00005	Loss of Allosteric site at R293	0.3	4.30E-03	Highly conserved, exposed and functional residue
6	R293H	ELME000053, ELME000062, ELME000064, ELME000147, ELME000220, PS00005	Loss of Allosteric site at R293	0.28	6.20E-03	Highly conserved, exposed and functional residue
			Altered Metal binding	0.19	0.03	
7	F304L	ELME000122, ELME000336	None			Average conserved, and buried residue
8	F304V	ELME000122, ELME000336	None			Average conserved, and buried residue
9	G422R	None	Gain of Strand	0.26	0.04	Highly conserved, exposed and functional residue
			Loss of Sulfation at Y417	0.02	0.03	
10	G433E	ELME000147, ELME000335, PS00008	Altered Transmembrane protein	0.1	0.05	Highly conserved, exposed and functional residue
			Gain of N-linked glycosylation at N437	0.05	0.02	
11	A520T	ELME000051, ELME000052, ELME000062, ELME000085, ELME000173, ELME000233, ELME000335	Loss of Loop	0.29	9.80E-03	Average conserved, buried and structural residue

### 3.5 Comparative structure modeling and validation of mutant PLK1 proteins

The mutated PLK1 structures were modelled using PyMol software after introducing residues of interest, including R175P, R175Q, L188P, L244F, R293C, R293H, F304L, F304V, G422R, G433E, and A520T. Then, the RMSD was calculated for 11 mutant PLK1 structures using the TM-align algorithm. This software is open-source and utilizes the TM-score rotation matrix to measure the RMSD value, determining the structural alignment between wild-type and mutant PLK1 proteins. The highest alignment represented the better structural similarity observed between mutant and wild-type PLK1. The amino acid properties of mutant PLK1 were examined through the HOPE server. It revealed the substitutions’ unfavorable changes in residue size, charge, or hydrophobicity, thereby disrupting the protein core and overall folding. The mutant R175P/Q induces loss of positive charge, as it is located in buried regions, reduces residue size, and disrupts hydrogen bonding. Similarly, L188P and F304 L/V were predicted as smaller residues than PLK1, which generate cavities inside the core and destabilize the protein structure. L244F causes steric clashes as they introduce bulkier residue in a buried environment. Critical mutations such as R293 C/H reduced residue size, caused cavity formation, altered hydrophobicity, and lost hydrogen bonds, which were essential for protein folding. Substitutions such as G422R and G433E introduced large and charged residues in a buried environment, which caused steric hindrances and altered backbone conformations. The mutation A520T also introduced larger and more polar residues in the protein core, which caused loss of hydrophobic interactions and might cause protein folding. Table 6 shows the structural alignment score, and amino acid properties of mutations. These results provided details on major amino acid properties such as size, charge, hydrophobicity, and torsion angles, along with the effects of amino acid substitutions on the structure and function of PLK1.

**TABLE 6:** Amino acid properties of mutations and structural alignment score predicted through HOPE and TM-align tools.

S. no	Amino acid change	Schematic structures of amino acid changes	Amino acid properties	TM alignment score
1	R175P	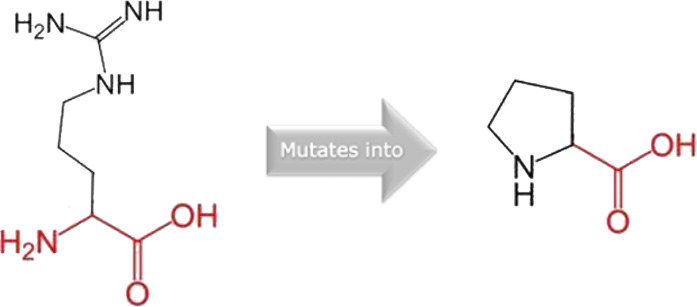	The mutant residue smaller than wild-type residue	0.997
			The charge of the buried wild-type residue is lost by this mutation	
			The mutation will cause an empty space in the core of the protein	
			The hydrophobicity of the wild-type and mutant residue differs	
			The mutation will cause loss of hydrogen bonds in the core of the protein and as a result disturb correct folding	
2	R175Q	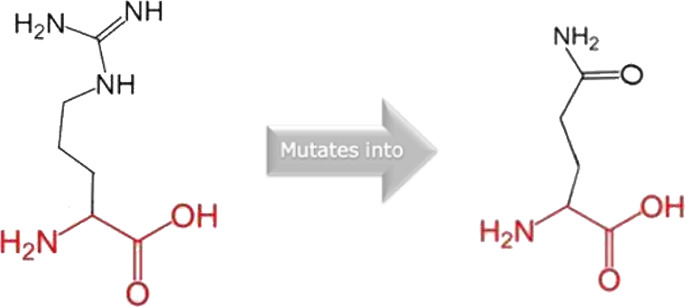	There is a difference in charge between wild-type and mutant amino acid	0.997
			The charge of the buried wild-type residue is lost by this mutation	
			The wild-type and mutant amino acids differ in size	
			The mutant residue smaller than wild-type residue	
			The mutation will cause an empty space in the core of the protein	
3	L188P	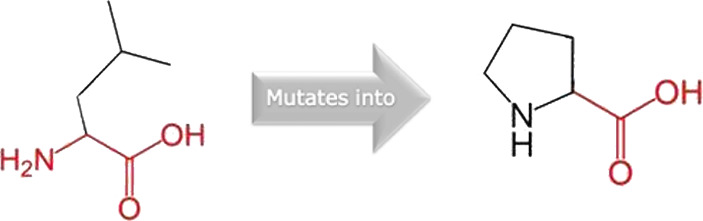	The wild-type and mutant amino acids differ in size	0.997
			The mutant residue smaller than wild-type residue	
			The mutation will cause an empty space in the core of the protein	
4	L244F	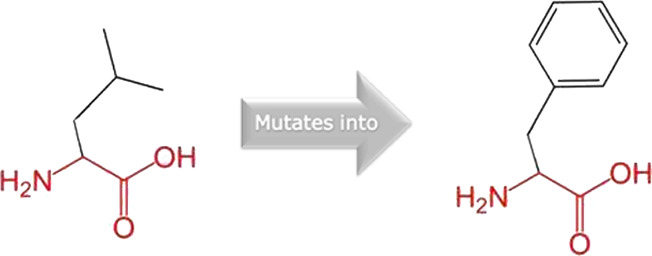	The wild-type and mutant amino acids differ in size	0.997
			The mutant residue is bigger than the wild-type residue	
			The wild-type residue was buried in the core of the protein. The mutant residue is bigger and probably will not fit	
5	R293C	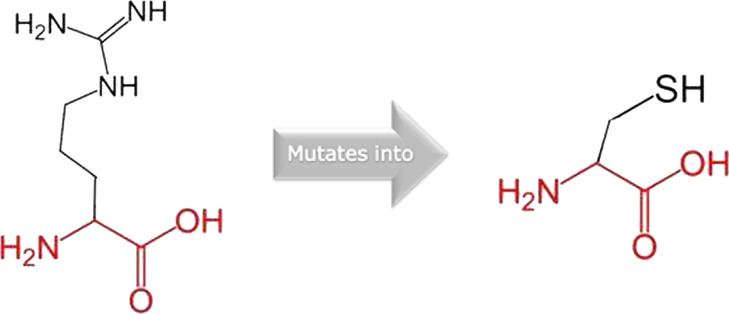	There is a difference in charge between wild-type and mutant amino acid	0.997
			The charge of the buried wild-type residue is lost by this mutation	
			The wild-type and mutant amino acids differ in size	
			The mutant residue smaller than wild-type residue	
			The mutation will cause an empty space in the core of the protein	
			The hydrophobicity of the wild-type and mutant residue differs	
			The mutation will cause loss of hydrogen bonds in the core of the protein and as a result disturb correct folding	
6	R293H	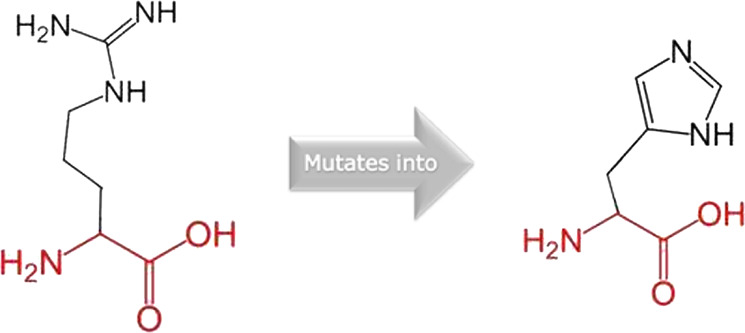	There is a difference in charge between wild-type and mutant amino acid	0.997
			The charge of the buried wild-type residue is lost by this mutation	
			The wild-type and mutant amino acids differ in size	
			The mutant residue is smaller than the wild-type residue	
			The mutation will cause an empty space in the core of the protein	
7	F304L	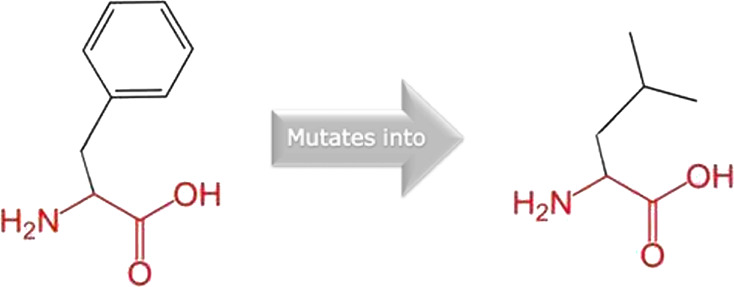	The wild-type and mutant amino acids differ in size	0.997
			The mutant residue is smaller than the wild-type residue	
			The mutation will cause an empty space in the core of the protein	
8	F304V	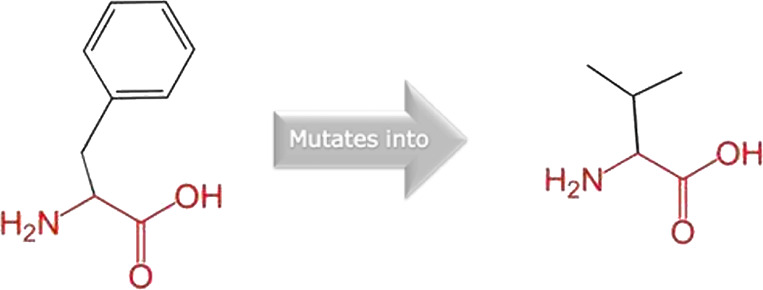	The wild-type and mutant amino acids differ in size	
			The mutant residue is smaller than the wild-type residue	
			The mutation will cause an empty space in the core of the protein	
9	G422R	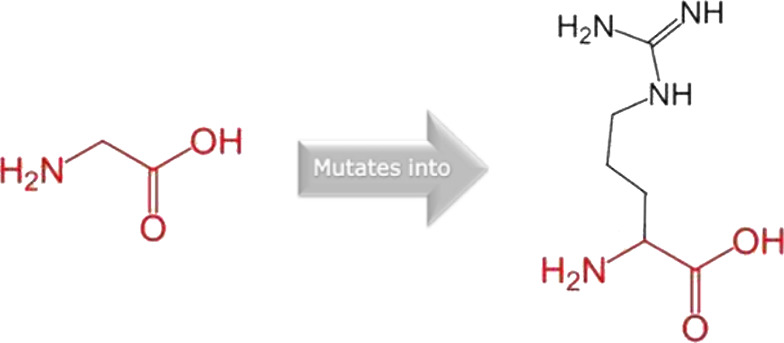	There is a difference in charge between wild-type and mutant amino acid	0.997
			The mutant residue introduces a charge in a buried residue which can lead to protein folding problems	
			The wild-type and mutant amino acids differ in size	
			The mutant residue is bigger than the wild-type residue	
			The wild-type residue was buried in the core of the protein. The mutant residue is bigger and probably will not fit	
			The torsion angles for this residue are unusual. Only glycine is flexible enough to make these torsion angles, mutation into another residue will force the local backbone into an incorrect conformation and will disturb the local structure	
10	G433E	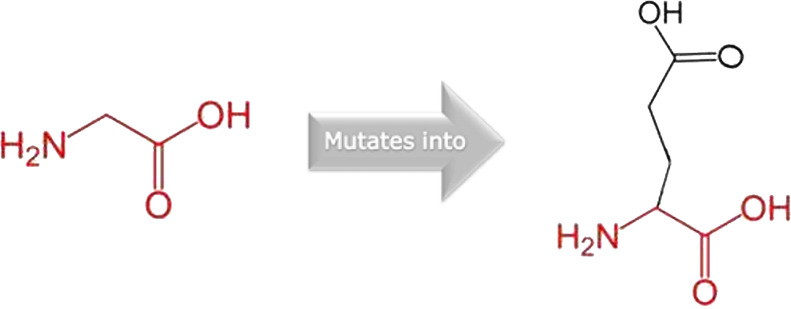	There is a difference in charge between wild-type and mutant amino acid	0.997
			The mutant residue introduces a charge in a buried residue which can lead to protein folding problems	
			The wild-type and mutant amino acids differ in size	
			The mutant residue is bigger than the wild-type residue	
			The wild-type residue was buried in the core of the protein. The mutant residue is bigger and probably will not fit	
			The torsion angles for this residue are unusual. Only glycine is flexible enough to make these torsion angles, mutation into another residue will force the local backbone into an incorrect conformation and will disturb the local structure	
11	A520T	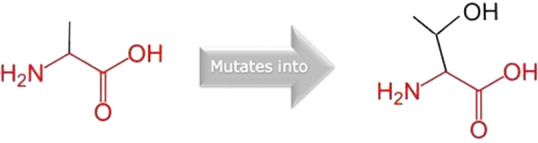	The wild-type and mutant amino acids differ in size	0.997
			The mutant residue is bigger than the wild-type residue	
			The wild-type residue was buried in the core of the protein. The mutant residue is bigger and probably will not fit	
			The hydrophobicity of the wild-type and mutant residue differs	
			The mutation will cause loss of hydrophobic interactions in the core of the protein	

### 3.6 Assessment of relative surface accessibility and secondary structure elements in mutant residues

The structural insights for PLK1 including relative surface accessibility (RSA), absolute surface accessibility (ASA), probability of disorder, and alpha-helix, beta-sheet, and coil formations, as well as information on residue types and secondary structure elements, were obtained using the NetSurf 3.0 server. The structural insights and relative surface accessibility of residues in PLK1 shown in Table 7. Graphical representation of wild-type PLK1’s secondary structure, relative surface accessibility and disorders are shown in Supplementary Figure S2. This server is recognized for its rapid, reliable, and accurate prediction of a protein’s structural features. The residues with high RSA values were significantly exposed to the solvent, while low RSA represented the residues buried in the core of the protein. Through analysis, some of the PLK1 mutants altered the surface accessibility, local conformation, and backbone geometry, which could impact structural stability and interaction properties. At residue R175, the amino acid substitutions with proline/glutamine maintained similar RSA but altered ASA and torsion angles, suggesting local flexibility changes, whereas the buried residues L188 and L244 mutated to proline and phenylalanine cause a shift to helix/coil conformations with reduced accessibility, which may destabilize the protein core. The mutations exposed to the R293 residue to cysteine/histidine established moderate RSA variations, whereasthe mutations F304 L/V decreased accessibility in the helix region, which reinforced the buried hydrophobic environment. Meanwhile, the glycine substitutions, such as G422R and G433E, significantly increased RSA and altered torsion angles, which might introduce bulky charged residues and disrupt local beta sheet arrangements. Collectively, these results suggested that the buried residues may alter the core protein by destabilizing it, while exposed residues may modulate the interactions and surface binding. Result from this analysis facilitates identification of potential drug targets, comprehension of protein-protein interactions, aid in the design of targeted molecules, and help in prioritizing proteins for experimental structure determination.

**TABLE 7:** Structural insights and relative surface accessibility of residues in PLK1 predicted through NetSurf 3.0

Wild-type amino acid	Residue number and class	Secondary structure	Relative surface accessibility (RSA) (%)	Absolute surface accessibility (Å)	Phi and Psi (^0^)	Mutant amino acid	Secondary structure	Relative surface accessibility (RSA) (%)	Absolute surface accessibility (ASA) (Å)	Phi and Psi (^0^)
R	175 and Exposed	Coil and turn	23	63	−4 and4	P	Coil and turn	23	36	−18 and −1
R	175 and Exposed	Coil and turn	23	63	−4 and4	Q	Coil and turn	24	54	−7 and3
L	188 and Buried	Coil and 3_10_ helix	6	13	−76 and −12	P	Coil and bend	6	10	67 and 31
L	244 and Buried	Helix and alpha helix	10	21	−74 and −30	F	Helix and alpha helix	3	6	−67 and −40
R	293 and Exposed	Coil and coil	12	33	−86 and 136	C	Coil and coil	13	22	−86 and 135
R	293 and Exposed	Coil and coil	12	33	−86 and 136	H	Coil and coil	15	33	−85 and 136
F	304 and Buried	Helix and 3_10_ helix	2	4	−63 and −38	L	Helix and 3_10_ helix	1	3	−63 and −37
F	304 and Buried	Helix and 3_10_ helix	2	4	−63 and −38	V	Helix and 3_10_ helix	1	2	−63 and −38
G	422 and Exposed	Coil and beta sheet	5	5	121 and 166	R	Coil and beta sheet	7	19	124 and 161
G	433 and Exposed	Strand and beta sheet	3	3	−125 and 149	E	Strand and beta sheet	4	9	−122 and 147
A	520 and Buried	Strand and beta sheet	3	4	−129 and 147	T	Strand and beta sheet	3	5	−130 and 148

### 3.7 Analysis of PPI network and effects of mutations

**FIGURE 8:**
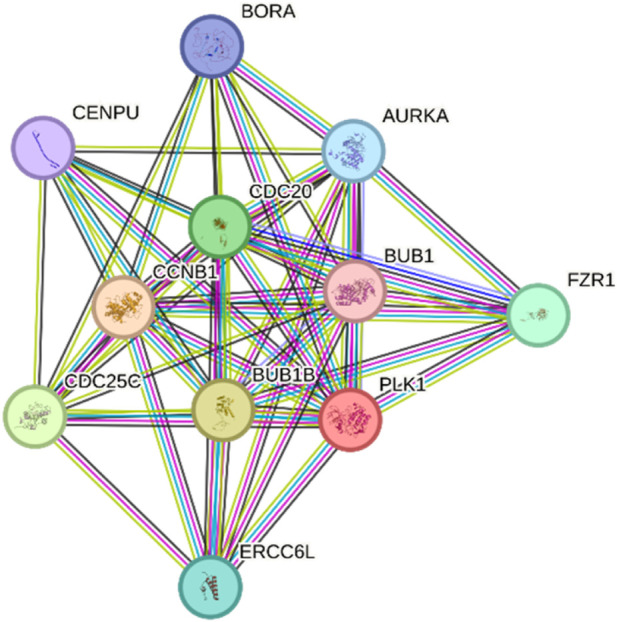
Protein-protein interactions of PLK1 predicted through STRING database.

The STRING database predicted a network of proteins that interact with the PLK1 protein, which determined 11 nodes and 48 edges. The nodes and edges represent the proteins and correlated proteins that interact with PLK1. High-confidence interactions with PLK1 included the proteins CCNB1, BUB1B, CDC25C, CDC20, FZR1, ERCC6L, AURKA, BORA, CENPU, and BUB1. Figure 8 shows the protein-protein interaction network of PLK1, while all the interactions are shown in Supplementary Table S5. The PPI network greatly regulates various biological processes, including the regulation of nuclear division, mitotic nuclear division, mitotic sister chromatid segregation, and anaphase-promoting complex-dependent catabolic processes. The molecular functions encompass anaphase-promoting complex binding, ubiquitin ligase activator activity, protein kinase binding, and protein serine kinase activity. The cellular components involved include the kinetochore, outer kinetochore, anaphase-promoting complex, mitotic checkpoint complex, and spindle. According to KEGG pathway analysis, the PP1 network is primarily involved in cell cycle pathways, progesterone-mediated oocyte maturation, and oocyte meiosis. The Reactome pathway demonstrated that the PPI network plays a significant role in activating various pathways, including the phosphorylation of EML1, APC/C-mediated degradation of cell cycle proteins, the resolution of sister chromatid cohesion, amplification of the signal from unattached kinetochores via a MAD2 inhibitory signal, and EML4 and NUDC in mitotic spindle formation. This functional enrichment analysis indicates that nsSNPs not only impact the function of PLK1 but also affect its key partners and numerous biochemical pathways, thereby significantly disrupting protein networks.

SAMMBE-3D predictions revealed that most of the PLK1 variants were likely to destabilize PPI interactions, and the results were validated through protein-protein docking using the ClusPro tool. Through using a cutoff value of DDG >0.5 kcal/mol, the destabilizing variants were determined, which include R175P (1.63 kcal/mol), R175Q (1.12 kcal/mol), L188P (1.02 kcal/mol), R293H (1.01 kcal/mol), R293C (0.91 kcal/mol), F304L (0.76 kcal/mol), and F304V (0.74 kcal/mol). These mutations were primarily located on the kinase N-lobe, substrate-binding region, and kinase domain (KD)-polo box domain (PBD) interface, suggesting potential impairment of catalytic activity and disruption of regulatory PPI partners such as CDC20 and ERCC6L. Further, the core mutations of PBD, such as G422R (0.07 kcal/mol) and G433E (0.26 kcal/mol), were found below the threshold value, suggesting they moderately destabilize the PPI network partners such as ERCC6L, BUB1B, and CENPU and possibly affect substrate recognition and mitotic regulation. In contrast, L244F (−0.13 kcal/mol) and A520T (−0.51 kcal/mol) were predicted to stabilize the PPI interactions moderately. Among them, the A520T mutation in the C-terminal region engaged in hydrogen bonding with BUB1B, suggesting it potentially disrupts PPI networks and affects overall protein stability. These results evidenced that PLK1 variants differentially impact protein stability and protein-interacting networks, with kinase-core mutations affecting enzymatic activity and PBD mutations modulating PPIs essential for cell cycle regulations. Effects of amino acid mutations on PPI interactions predicted through SAAMBE 3D and PBDsum servers are shown in Table 8. The binding affinity and number of interacting residues of PPI networks predicted through ClusPro and PDBsum are given in Supplementary Table S6. The protein-protein interactions of PLK1 with partners proteins are given in Supplementary Figure S3.

**TABLE 8:** Effects of amino acid mutations on PPI interactions predicted through SAAMBE 3D and PDBsum.

PLK1 mutation	Domain location	Binding free energy (DDG-kcal/mol)/Stability	Interacting proteins (from STRING)	Details of PPI interface	Potential impact on PPIs and function
R175P/Q	Kinase N-lobe (catalytic core)	1.63/1.12 (destabilizing)	None directly linked	Not in interface	Likely affects kinase folding and catalytic efficiency; may reduce ATP/substrate binding indirectly
L188P	Kinase Domain (KD)	1.02 (destabilizing)	None directly linked	Near substrate-binding surface	May impair substrate binding and kinase activity due to local structural distortion
L244F	Kinase Domain (C-lobe)	−0.13 (slightly stabilizing)	None directly linked	Not in the active site	Minimal impact on function; slight stabilization unlikely to affect catalysis or PPI.
R293 C/H	KD–PBD linker	0.91/1.01 (destabilizing)	ERCC6L, FZR1	At interface between KD-PBD; near Glu303 of ERCC6L interface	Could weaken KD-PBD interaction, altering substrate specificity or PPI with ERCC6L
F304 L/V	KD–PBD interface	0.76/0.74 (destabilizing)	CDC20, ERCC6L	Adjacent to KD-PBD interaction surface; near Ser306 of CDC20 interface	Likely affects KD-PBD structural integrity; may reduce CDC20/ERCC6L binding, affecting cell cycle regulation
G422R	Polo-box Domain (PBD core)	0.07 (destabilizing)	ERCC6L, BUB1B, BORA, BUB1	Close to peptide binding cleft (TRP414 and TYR417); near Tyr421 of ERCC6L interface	Could perturb PBD peptide recognition, impairing recruitment of PBD-dependent substrates or regulators
G433E	PBD (core)	0.26 (destabilizing)	ERCC6L, CENPU	Near groove stabilizing loop; near Leu435 of ERCC6L interface	May disrupt structural loop important for PBD stability; potential moderate effect on PPI with ERCC6L/CENPU.
A520T	PBD (C-terminal portion)	−0.51 (stabilizing)	BUB1B, CENPU	In interface with BUB1B (H520 forms H-bond with L208)	Could enhance PBD-BUB1B interaction; may affect timing of mitotic checkpoint signalling

Where, DDG-Binding free energy, KD-Kinase domain, PBD-Polo box domain.

### 3.8 Analysis of post translational modifications of PLK1 mutants

**FIGURE 9:**
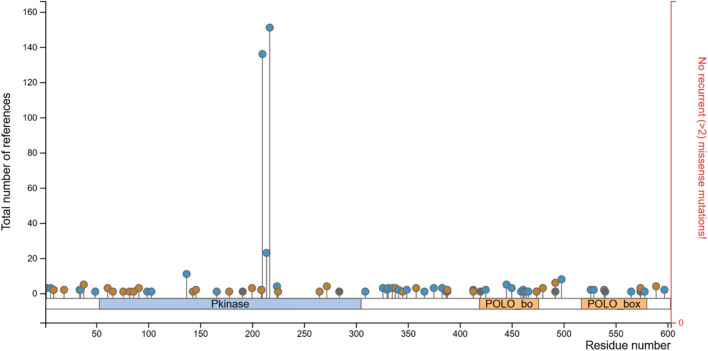
Experimentally determined PTM of PLK1 protein predicted through PhosphoSitePlus.

The amino acid mutation effects on PTM sites were predicted through MusiteDeep and PhosphoSite plus tools. The predictions from MusiteDeep with a probability threshold of greater than 0.5 for general PTMs and greater than 0.7 for phosphorylation sites showed no gain or loss of major PTM motifs across all variants. A cross-check with the PhosphoSitePlus database revealed that none of the affected wild-type residues were annotated as PTM sites in PLK1 proteins. Figure 9 showcases the experimentally determined PTM sites using high- and low-throughput papers, where the red line represents that no somatic mutations are present in the PLK1’s PTM sites. However, the molecular mechanism from MutPred 2 suggested that some amino acid substitutions indirectly influenced PTM potential. For instance, G422R was associated with predicted loss of sulfation at Y417, while G433E was linked to N-linked glycosylation at N437. Similarly, R293 C/H and L244F were predicted to alter local structural motifs, which may interfere with protein binding. Taking this into account, the direct PTM distribution is unlikely, and certain variants may subtly affect PLK1 regulation through altering structural features or accessibility for docking and regulatory motifs rather than direct loss of PTM sites.

### 3.9 Structural stability evaluation of native and mutant PLK1 proteins

The structural stabilities of wild-type PLK1 and mutant PLK1, including R175P (A), R175Q (B), L188P (C), L244F (D), R293C (E), R293H (F), F304L (G), F304V (H), G422R (I), G433E (J), and A520T (K) along with wild-type PLK1 were predicted through MDS. Mutants A to H are located in the kinase domain, while mutants I to K are located in the polo-box domains. The key structural parameters, such as RMSD, RMSF, SASA, and ROG, for both wild-type PLK1 (black color) and mutant PLK1s (red color) were analyzed over the trajectory of 100 ns.

**FIGURE 10:**
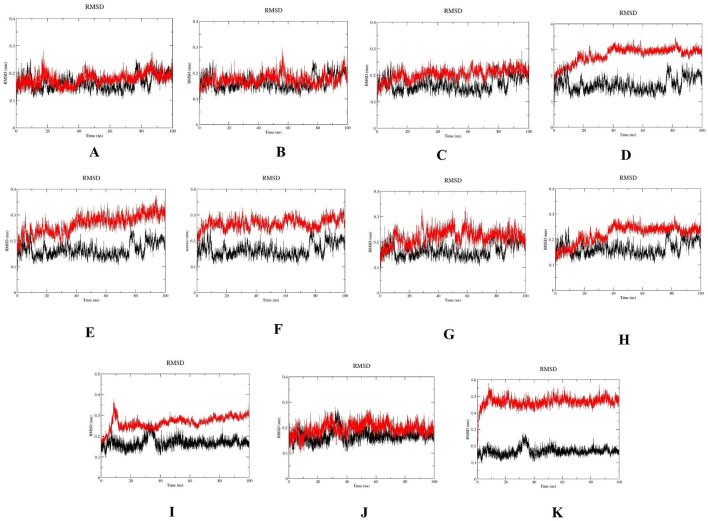
Root mean square deviation (RMSD) plots for wild-type PLK1 (black) and mutant PLK1s (red), where **(A)** R175P, **(B)** R175Q, **(C)** L188P, **(D)** L244F, **(E)** R293C, **(F)** R293H, **(G)** F304L, **(H)** F304V, **(I)** G422R, **(J)** G433E, and **(K)** A520T.

RMSD trajectories reflect the overall stability of protein backbones (Sinha et al., 2022a). The wild-type PLK1 exhibited an average RMSD ranging from 0.10 to 0.25 nm, indicating stable conformational dynamics. The mutants L244F, R293C, and F304L had higher RMSD values, between 0.25 and 0.25 nm, indicating less stability and more structural changes. The mutants R175P, R175Q, L188P, and F304V exhibited RMSD around 0.15–0.28 nm, indicating less structural stability. Polo box domain mutations (I and J) showed stable RMSD around 0.2–0.25 nm, while A520T showed stable RMSD around 0.4–0.55 nm and deviated from wild-type PLK1, suggesting structural destabilization. Figure 10 represents the RMSD of wild-type PLK1 and mutant PLK1s.

**FIGURE 11:**
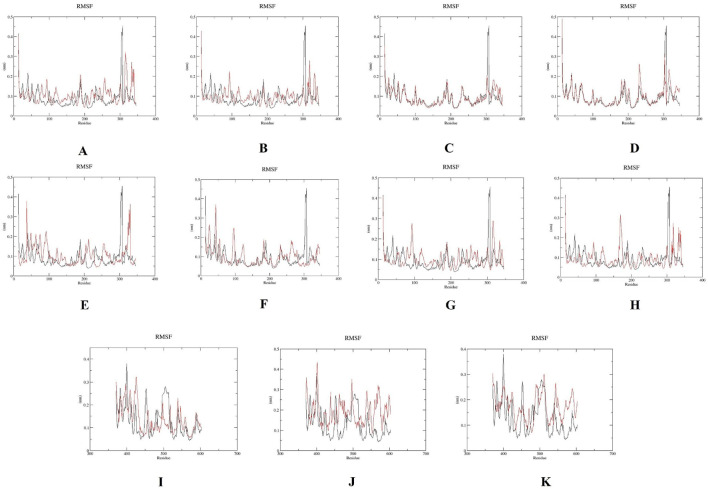
Root mean square fluctuations (RMSF) plots for wild-type PLK1 (black) and mutant PLK1s (red), where **(A)** R175P, **(B)** R175Q, **(C)** L188P, **(D)** L244F, **(E)** R293C, **(F)** R293H, **(G)** F304L, **(H)** F304V, **(I)** G422R, **(J)** G433E, and **(K)** A520T.

The per-residue flexibility of the mutant and wild-type PLK1 structure was evaluated through RMSF. The RMSF plots are shown in Figure 11. The wild-type PLK1 residues fluctuated within 0.10–0.38 nm, with loop regions showing higher variability. Mutants present in the kinase domain (A to H) and polo-box domains (I to K) exhibited increased residue fluctuations exceeding 0.25 nm at specific positions, particularly near mutation sites and in loops. This finding confirms that the local flexibility might affect the functional domains.

**FIGURE 12:**
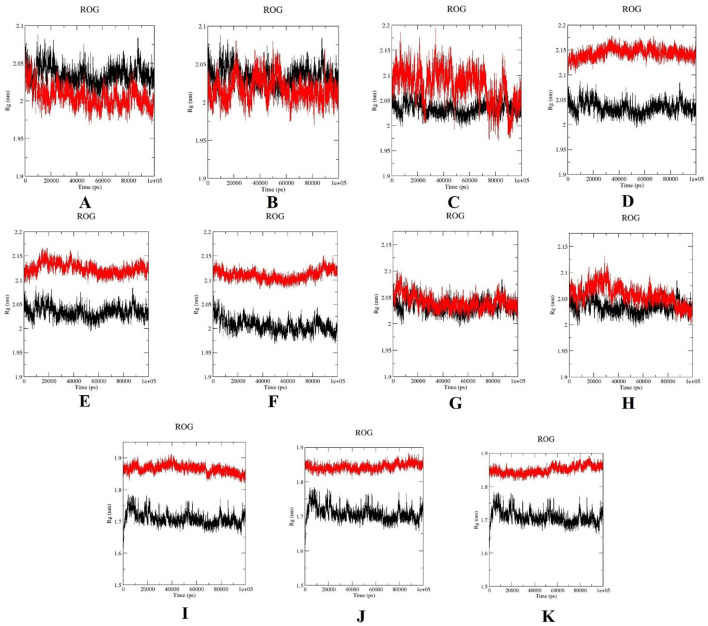
Radius of gyration (ROG) plots for wild-type PLK1 (black) and mutant PLK1s (red), where **(A)** R175P, **(B)** R175Q, **(C)** L188P, **(D)** L244F, **(E)** R293C, **(F)** R293H, **(G)** F304L, **(H)** F304V, **(I)** G422R, **(J)** G433E, and **(K)** A520T.

The protein’s compactness analysis through plotting ROG. From Figure 12, the wild-type PLK1 maintained a consistent ROG value around 2–2.07 nm (A to H) and 1.65 to 1.78 (I to K). The mutants L244F, R293C, and R293H showed increased ROG values, ranging from 2.1 to 2.17 nm; subsequently, the I to K mutations also showed elevated ROG values, ranging from 1.85 to 1.90 nm (Figure 12), suggesting a tendency towards structural loosening or expansion. Other PLK1 mutant structures have ROG values closely resembling wild-type PLK1, implying preserved global folding integrity.

**FIGURE 13:**
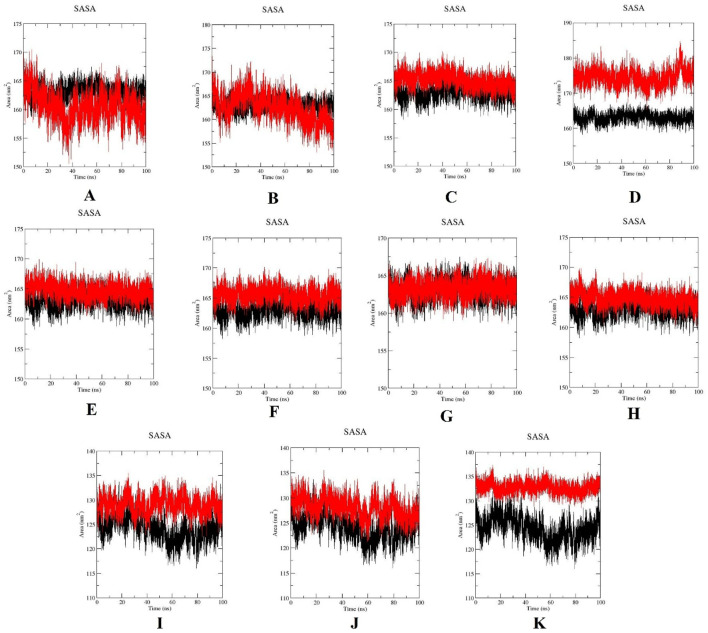
Solvent accessible surface area (SASA) plots for wild-type PLK1 (black) and mutant PLK1s (red), where **(A)** R175P, **(B)** R175Q, **(C)** L188P, **(D)** L244F, **(E)** R293C, **(F)** R293H, **(G)** F304L, **(H)** F304V, **(I)** G422R, **(J)** G433E, and **(K)** A520T.

The surface exposure to solvent was determined through SASA. The SASA graphs are shown in Figure 13. The increased solvent exposure was observed in L244F, ranging from 170 to 185 nm; subsequently, the high solvent exposure was discovered in the A520T mutation, ranging from 130 to 137 nm^2^. This observation indicates that greater surface exposure or partial unfolding occurs in the mutant structures. Conversely, the other mutant structures showed SASA values comparable to wild-type PLK1, suggesting retention of structural surface integrity.

**FIGURE 14:**
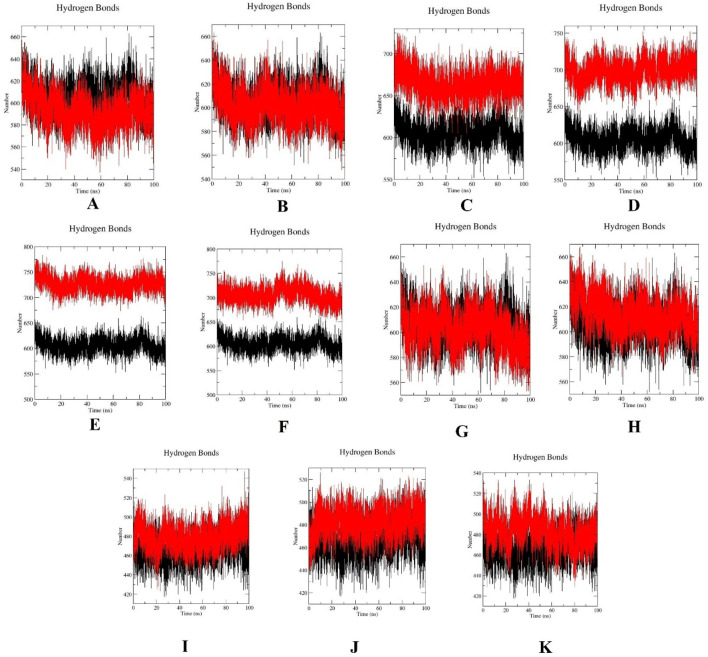
Hydrogen bond (H-bonds) plots for wild-type PLK1 (black) and mutant PLK1s (red), where **(A)** R175P, **(B)** R175Q, **(C)** L188P, **(D)** L244F, **(E)** R293C, **(F)** R293H, **(G)** F304L, **(H)** F304V, **(I)** G422R, **(J)** G433E, and **(K)** A520T.

The stability between wild-type PLK1 and mutant PLK1 structure was determined through H-bond analysis. Figure 14 shows H-bonds for wildtype-PLK1 and mutants PLK1s. Through analysis, the wild-type PLK1 in KD typically maintained H-bonds between ∼550 and 650 within the biological environment, whereas the mutants R175P/Q, L188P, and F304 L/V showed a consistently higher number of H-bonds (550–660). The subtle changes caused by proline mutations may break helices and alter local packing. On the other hand, the mutants such as L244F and R293 C/H exhibited the number of H-bonds between 650 and 750, suggesting these mutations stabilized local secondary structures and reduced mobility of the activation loop. Following that, the mutations present in PBD, such as G422R, G433E, and A520T, exhibited a moderately increased number of H-bonds (∼460–550) compared to wildtype PLK1 (420–510), reflecting on altered loop conformations and solvent exposure as indicated by SASA. This modification leads to loss of structural integrity and deviation from the native fold of the protein. These findings suggested that the structural rearrangement altered conformations through potentially impacting flexibility and catalytic activity.

Overall, the MDS indicated that the mutations L244F, R293C, and R293H in the kinase domain and A520T in the polo-box domain may perturb the conformational stability, flexibility, compactness, solvent accessibility of PLK1. Moreover, the other mutations, R175P, R175Q, L188P, F304L, F304V, G422R, and G433E, had a moderate impact on PLK1’s structural dynamics.

## 4 Discussion

A serine/threonine kinase protein, polo-like kinase 1, is responsible for cell cycle regulation and activation of various proteins that are involved in cellular processes. Despite being known for its potential among other PLK types (PLK2, PLK3, PLK4, and PLK5), PLK1 is determined recently as a potential oncogenic target across various cancer types due to its extensive research exploration and essential mitotic roles (Fang et al., 2022; Jiawei et al., 2022; Garlapati et al., 2023; Guerrero-Zotano et al., 2023). To establish the clinical relevance, the survival analysis determined that high expression of PLK1 was significantly correlated with poor OS and DFS in multiple cancers, notably lung and breast cancers. Following that, the oncogenic missense mutations were identified using computational approaches that greatly impact the functional and structural consequences of the PLK1 protein. A total of 207 nsSNP mutations of PLK1 were retrieved from cBioPortal by analyzing all types of cancers. Following that, various functional and structural prediction tools, such as SIFT, PolyPhen-2, E-SNPs and GO, MutPred2, CScape, FATHMM-XF, I-Mutant 2.0, CUPSAT, DynaMut2, and mCSM. SIFT were utilized to screen deleterious, damaging, pathogenic, and destabilizing nsSNPs. These tools provide comprehensive annotations on variants by integrating data from various resources like genomic, protein, structural, and functional repositories (Dash et al., 2020; Dhakar et al., 2022; Ramayanam et al., 2022; Tastan Bishop et al., 2022). Through this analysis, a total of 8 mutations from the kinase domain (13-345 aa): R175P (A), R175Q (B), L188P (C), L244F (D), R293C (E), R293H (F), F304L (G), and F304V (H), and 3 mutations from the polo-box domain (371-603 aa): G422R (I), G433E (J), and A520T (K) were identified as potentially damaging and highly destabilizing mutations that impact PLK1 structure and functions. These mutations associated with specific types of cancer include bladder cancer (R175P), breast cancer (L244F), colon cancer (R293C, R293H, G422R, G433E, and A520T), colorectal cancer (R175Q and G422R), endometrial cancer (R175Q, L188P, F304V, and A520T), esophageal cancer (R293C), head and neck cancer (R293H), lung cancer (R293H), kidney cancer (R293C), skin cancer (A520T), and uterine cancer (R175Q, L188P, F304L, F304V, and A520T).

Following that, conservation profiles and RSA were determined for each amino acid position. The mutations at the 175, 293, 422, and 433 amino acid positions were found to be exposed and functional residues, whereas mutations in the 244 and 520 amino acid positions were determined to be buried and structural residues. Concurrently, the mutations and amino acid properties were predicted using the HOPE server, which suggested that the kinase domain mutations (A to H) more often created cavity formation, charge imbalances, or backbone distortion, leading to improper protein folding and affecting functional conformations, while the polo-box domain mutations (I to K) affected the structural rigidity and interaction surfaces, which are necessary for proper localization and substrate recognition. Further, the STRING database was used to investigate signalling events in human of interacting proteins that are linked to PLK1. This repository combines information from multiple public pathways and interaction databases, which is used effectively to query the proteins (Kamal et al., 2024). Through understanding protein-protein interactions and pathways, the effect of mutations and their roles in key biochemical processes were evaluated. Integrating PTM and PPI predictions suggested that the PLK1 mutations cause destabilization in PPI, which was analyzed through DDG profiles. By analyzing the results, this study found that several destabilizing variants overlapped with or were proximal to experimentally defined PPI interfaces such as CDC20, BUB1B, CENPU, and ERCC6L. Further, the PhosphoSitePlus database predicted that none of the residues corresponded to experimentally validated PTM sites. Nevertheless, MutPred 2 forecasted that several variants, such as R175P/Q, L244F, G422R, and G433E, were potentially associated with gain or loss of PTMs, which may disrupt local structural changes influencing accessibility and recognition by modifying enzymes. This analysis indicates that PLK1 is recognized as having high-priority structural and functional relationships with interacting proteins and facilitating drug-target identification.

In previous literature, the researchers identified potential nsSNPs of PLK1 from 24 missense SNPs that are associated with various cancer types. Previous work indicates that the W414F mutation in the polo-box domain reduces the structural stability and identified it as a potential target with the molecular dynamics simulation (MDS) conducted for 40 ns (Kamaraj et al., 2013). In order to capture the more extensive and conformational changes, this current research utilized a 100 ns simulation duration (Yadav and Singh, 2021). Through computational methods, rapid screening, hypothesis generation, and molecular-level mechanistic insights on oncogenic missense mutations in the PLK1 protein were examined (Sinha et al., 2022a; 2023). By analyzing the MDS results, the eight mutations presented in the kinase domain substantially affect the protein’s structural integrity. The evaluation parameters, such as elevated RMSD and RMSF, increased ROG, high exposure to solvent (SASA) and increase H-bonds were determined in mutations L244F, R293C, and R293H. This analysis demonstrated that these mutations have the capability to highly disrupt secondary structure elements, such as alpha helixes and beta sheets, resulting in the alteration of protein-protein interaction surfaces. They also disrupt the essential functional elements involved in ATP binding and phosphorylation. On the other hand, the mutations observed in polo-box domains, G422R and G433E, had minimal influence on structural destabilization, while A520T showcased high structural destabilization, which leads to affecting substrate recognition and potential functional implications.

All these findings highlighted the importance of PLK1 expression in different cancers and suggested it could be a potential target for cancer treatment. However, the confirmation of altered kinase activity, binding affinities, and cellular assays is necessary to validate the effects of the mutations on cell cycle progression, proliferation, and apoptosis. Furthermore, the investigation of PLK1’s potential as a prognostic biomarker should involve studies with larger cohorts. Additionally, the identification of specific-target drugs should focus on particular mutant forms of PLK1 to modulate the activity of cancer candidates, which aligns with the movement toward personalized medicinal approaches.

## 5 Conclusion

This study extensively identified and characterized deleterious missense mutations in PLK1 utilizing integrative bioinformatics and molecular dynamic simulation approaches. Initially, the survival outcomes validated that the clinical relevance of PLK1 overexpression is more highly associated with poor prognosis in breast and lung cancers than other types. Following that, structural and functional analysis prioritized 11 high-risk-associated nsSNPs with key mutations on the kinase domain, such as L244F, R293C, and R293H, and the polo-box domain such as A520T, which may perturb the protein structure, stability, and function. Concurrently, the mechanistic insights of each amino acid mutation determined that the missense variants significantly affect the control of the cell cycle and promote tumor growth. The findings from this study established that PLK1 is not only recognized as a critical multi-target oncogene but also as a viable target for cancer diagnostics and therapeutics. In the future, the perspective of functional impacts of these mutations should be validated in *in vitro* and *in vivo* conditions, which enhance the exploration of identifying mutation-specific therapeutic strategies to support precision oncology.

## Data Availability

The raw data supporting the conclusions of this article will be made available by the authors, without undue reservation.

## References

[B1] Abdalla M. Eltayb W. A. El-Arabey A. A. Singh K. Jiang X. (2022). Molecular dynamic study of SARS-CoV-2 with various S protein mutations and their effect on thermodynamic properties. Comput. Biol. Med. 141, 105025. 10.1016/j.compbiomed.2021.105025 34772510 PMC8576119

[B2] Adzhubei I. A. Schmidt S. Peshkin L. Ramensky V. E. Gerasimova A. Bork P. (2010). A method and server for predicting damaging missense mutations. Nat. Methods 7, 248–249. 10.1038/nmeth0410-248 20354512 PMC2855889

[B56] Alzahrani F. A. Ahmed F. Sharma M. Rehan M. Mahfuz M. Baeshen S. (2020). Investigating the pathogenic SNPs in BLM helicase and their biological consequences by computational approach. Sci. Rep. 10 (01), 12377. 10.1038/s41598-020-69033-8 32704157 PMC7378827

[B3] Ashkenazy H. Abadi S. Martz E. Chay O. Mayrose I. Pupko T. (2016). ConSurf 2016: an improved methodology to estimate and visualize evolutionary conservation in macromolecules. Nucleic Acids Res. 44, W344–W350. 10.1093/nar/gkw408 27166375 PMC4987940

[B4] Bray F. Laversanne M. Sung H. Ferlay J. Siegel R. L. Soerjomataram I. (2024). Global cancer statistics 2022: GLOBOCAN estimates of incidence and mortality worldwide for 36 cancers in 185 countries. CA Cancer J. Clin. 74, 229–263. 10.3322/caac.21834 38572751

[B5] Capriotti E. Fariselli P. Casadio R. (2005). I-Mutant2.0: predicting stability changes upon mutation from the protein sequence or structure. Nucleic Acids Res. 33, W306–W310. 10.1093/nar/gki375 15980478 PMC1160136

[B6] Chiappa M. Petrella S. Damia G. Broggini M. Guffanti F. Ricci F. (2022). Present and future perspective on PLK1 inhibition in cancer treatment. Front. Oncol. 12, 903016. 10.3389/fonc.2022.903016 35719948 PMC9201472

[B7] Dash R. Ali M. C. Rana M. L. Munni Y. A. Barua L. Jahan I. (2020). Computational SNP analysis and molecular simulation revealed the Most deleterious missense variants in the NBD1 domain of human ABCA1 transporter. Int. J. Mol. Sci. 21, 7606–7623. 10.3390/ijms21207606 33066695 PMC7589834

[B8] Dhakar R. Dakal T. C. Sharma A. (2022). Genetic determinants of lung cancer: understanding the oncogenic potential of somatic missense mutations. Genomics 114, 110401. 10.1016/j.ygeno.2022.110401 35709927

[B9] Fang L. Liu Q. Cui H. Zheng Y. Wu C. (2022). Bioinformatics analysis highlight differentially expressed CCNB1 and PLK1 genes as potential anti-breast cancer drug targets and prognostic markers. Genes (Basel) 13, 654. 10.3390/genes13040654 35456460 PMC9027215

[B10] Gao J. Aksoy B. A. Dogrusoz U. Dresdner G. Gross B. Sumer S. O. (2013). Integrative analysis of complex cancer genomics and clinical profiles using the cBioPortal. Sci. Signal 6, pl1. 10.1126/scisignal.2004088 23550210 PMC4160307

[B11] Garlapati C. Joshi S. Bhattarai S. Krishnamurthy J. Turaga R. C. Nguyen T. (2023). PLK1 and AURKB phosphorylate survivin differentially to affect proliferation in racially distinct triple-negative breast cancer. Cell Death Dis. 14, 12. 10.1038/s41419-022-05539-5 36627281 PMC9832024

[B12] Guerrero-Zotano Á. Belli S. Zielinski C. Gil-Gil M. Fernandez-Serra A. Ruiz-Borrego M. (2023). CCNE1 and PLK1 mediate resistance to palbociclib in HR+/HER2- metastatic breast cancer. Clin. Cancer Res. 29, 1557–1568. 10.1158/1078-0432.CCR-22-2206 36749874 PMC10102847

[B13] Győrffy B. (2024). Integrated analysis of public datasets for the discovery and validation of survival-associated genes in solid tumors. Innovation 5, 100625. 10.1016/j.xinn.2024.100625 38706955 PMC11066458

[B14] Høie M. H. Kiehl E. N. Petersen B. Nielsen M. Winther O. Nielsen H. (2022). NetSurfP-3.0: accurate and fast prediction of protein structural features by protein language models and deep learning. Nucleic Acids Res. 50, W510–W515. 10.1093/nar/gkac439 35648435 PMC9252760

[B15] Hornbeck P. V. Zhang B. Murray B. Kornhauser J. M. Latham V. Skrzypek E. (2015). PhosphoSitePlus, 2014: mutations, PTMs and recalibrations. Nucleic Acids Res. 43, D512–D520. 10.1093/nar/gku1267 25514926 PMC4383998

[B16] Jiawei W. Xiajun B. Tian S. Xuzheng G. Zhenwang Z. (2022). Comprehensive analysis of PLKs expression and prognosis in breast cancer. Cancer Genet. 268, 83–92. 10.1016/j.cancergen.2022.09.007 36206661

[B17] Jones G. Jindal A. Ghani U. Kotelnikov S. Egbert M. Hashemi N. (2022). Elucidation of protein function using computational docking and hotspot analysis by ClusPro and FTMap. Acta Crystallogr. D. Struct. Biol. 78, 690–697. 10.1107/S2059798322002741 35647916 PMC9159284

[B18] Kahl I. Mense J. Finke C. Boller A. L. Lorber C. Győrffy B. (2022). The cell cycle-related genes RHAMM, AURKA, TPX2, PLK1, and PLK4 are associated with the poor prognosis of breast cancer patients. J. Cell Biochem. 123, 581–600. 10.1002/jcb.30205 35014077

[B19] Kamal M. M. Mia M. S. Faruque M. O. Rabby M. G. Islam M. N. Talukder M. E. K. (2024). *In silico* functional, structural and pathogenicity analysis of missense single nucleotide polymorphisms in human MCM6 gene. Sci. Rep. 14, 11607. 10.1038/s41598-024-62299-2 38773180 PMC11109216

[B20] Kamaraj B. Rajendran V. Sethumadhavan R. Purohit R. (2013). *In-silico* screening of cancer associated mutation on PLK1 protein and its structural consequences. J. Mol. Model 19, 5587–5599. 10.1007/s00894-013-2044-0 24271645

[B21] Kandala S. Ramos M. Voith von Voithenberg L. Diaz-Jimenez A. Chocarro S. Keding J. (2023). Chronic chromosome instability induced by Plk1 results in immune suppression in breast cancer. Cell Rep. 42, 113266. 10.1016/j.celrep.2023.113266 37979172

[B22] Khan A. S. Akter M. Enni M. A. Khan S. F. (2022). An *in silico* approach for the identification of detrimental missense SNPs and their potential impacts on human CRY2 protein. 10.21203/rs.3.rs-2400566/v1

[B23] Laskowski R. A. (2022). *PDBsum1*: a standalone program for generating PDBsum analyses. Protein Sci. 31, e4473. 10.1002/pro.4473 36251626 PMC9667822

[B24] Lemkul J. (2019). From proteins to perturbed hamiltonians: a suite of tutorials for the GROMACS-2018 molecular simulation package [article v1.0]. Living J. Comput. Mol. Sci. 1. 10.33011/livecoms.1.1.5068

[B25] Lim J. Hwang Y. S. Yoon H. R. Yoo J. Yoon S. R. Jung H. (2024). PLK1 phosphorylates RhoGDI1 and promotes cancer cell migration and invasion. Cancer Cell Int. 24, 73. 10.1186/s12935-024-03254-z 38355643 PMC10865702

[B26] Manfredi M. Savojardo C. Martelli P. L. Casadio R. (2022). E-SNPs&GO: embedding of protein sequence and function improves the annotation of human pathogenic variants. Bioinformatics 38, 5168–5174. 10.1093/bioinformatics/btac678 36227117 PMC9710551

[B27] Navapour L. Mogharrab N. (2021). *In silico* screening and analysis of nonsynonymous SNPs in human CYP1A2 to assess possible associations with pathogenicity and cancer susceptibility. Sci. Rep. 11, 4977. 10.1038/s41598-021-83696-x 33654112 PMC7925555

[B28] Ng P. C. (2003). SIFT: predicting amino acid changes that affect protein function. Nucleic Acids Res. 31, 3812–3814. 10.1093/nar/gkg509 12824425 PMC168916

[B29] Pahari S. Li G. Murthy A. K. Liang S. Fragoza R. Yu H. (2020). SAAMBE-3D: predicting effect of mutations on protein–protein interactions. Int. J. Mol. Sci. 21, 2563. 10.3390/ijms21072563 32272725 PMC7177817

[B30] Parthiban V. Gromiha M. M. Schomburg D. (2006). CUPSAT: prediction of protein stability upon point mutations. Nucleic Acids Res. 34, W239–W242. 10.1093/nar/gkl190 16845001 PMC1538884

[B31] Pejaver V. Urresti J. Lugo-Martinez J. Pagel K. A. Lin G. N. Nam H.-J. (2020). Inferring the molecular and phenotypic impact of amino acid variants with MutPred2. Nat. Commun. 11, 5918. 10.1038/s41467-020-19669-x 33219223 PMC7680112

[B32] Pires D. E. V. Ascher D. B. Blundell T. L. (2014a). DUET: a server for predicting effects of mutations on protein stability using an integrated computational approach. Nucleic Acids Res. 42, W314–W319. 10.1093/nar/gku411 24829462 PMC4086143

[B33] Pires D. E. V. Ascher D. B. Blundell T. L. (2014b). mCSM: predicting the effects of mutations in proteins using graph-based signatures. Bioinformatics 30, 335–342. 10.1093/bioinformatics/btt691 24281696 PMC3904523

[B34] Ramayanam N. R. Manickam R. Mahalingam V. T. Goh K. W. Ardianto C. Ganesan P. (2022). Functional and structural impact of deleterious missense single nucleotide polymorphisms in the NR3C1, CYP3A5, and TNF-α genes: an *in silico* analysis. Biomolecules 12, 1307. 10.3390/biom12091307 36139147 PMC9496109

[B35] Rodrigues C. H. M. Pires D. E. V. Ascher D. B. (2021). DynaMut2: assessing changes in stability and flexibility upon single and multiple point missense mutations. Protein Sci. 30, 60–69. 10.1002/pro.3942 32881105 PMC7737773

[B36] Rogers M. F. Shihab H. A. Gaunt T. R. Campbell C. (2017). CScape: a tool for predicting oncogenic single-point mutations in the cancer genome. Sci. Rep. 7, 11597. 10.1038/s41598-017-11746-4 28912487 PMC5599557

[B37] Rogers M. F. Shihab H. A. Mort M. Cooper D. N. Gaunt T. R. Campbell C. (2018). FATHMM-XF: accurate prediction of pathogenic point mutations *via* extended features. Bioinformatics 34, 511–513. 10.1093/bioinformatics/btx536 28968714 PMC5860356

[B38] Sathishkumar K. Chaturvedi M. Das P. Stephen S. Mathur P. (2022). Cancer incidence estimates for 2022 and projection for 2025: result from national cancer registry programme, India. Indian J. Med. Res. 156, 598–607. 10.4103/ijmr.ijmr_1821_22 36510887 PMC10231735

[B39] Sinha S. Wang S. M. (2020). Classification of VUS and unclassified variants in BRCA1 BRCT repeats by molecular dynamics simulation. Comput. Struct. Biotechnol. J. 18, 723–736. 10.1016/j.csbj.2020.03.013 32257056 PMC7125325

[B40] Sinha S. Qin Z. Tam B. Wang S. M. (2022a). Identification of deleterious variants of uncertain significance in BRCA2 BRC4 repeat through molecular dynamics simulations. Brief. Funct. Genomics 21, 202–215. 10.1093/bfgp/elac003 35325018

[B41] Sinha S. Tam B. Wang S. M. (2022b). Applications of molecular dynamics simulation in protein study. Membr. (Basel) 12, 844. 10.3390/membranes12090844 36135863 PMC9505860

[B42] Sinha S. Li J. Tam B. Wang S. M. (2023). Classification of PTEN missense VUS through exascale simulations. Brief. Bioinform 24, bbad361. 10.1093/bib/bbad361 37843401

[B43] Stephenson J. D. Totoo P. Burke D. F. Jänes J. Beltrao P. Martin M. J. (2024). ProtVar: mapping and contextualizing human missense variation. Nucleic Acids Res. 52, W140–W147. 10.1093/nar/gkae413 38769064 PMC11223857

[B44] Su S. Chhabra G. Singh C. K. Ndiaye M. A. Ahmad N. (2022). PLK1 inhibition-based combination therapies for cancer management. Transl. Oncol. 16, 101332. 10.1016/j.tranon.2021.101332 34973570 PMC8728518

[B45] Subramani N. K. Venugopal S. (2024). Molecular docking and dynamic simulation studies of bioactive compounds from traditional medicinal compounds against exfoliative toxin B from *Staphylococcus aureus* . J. Pharmacol. Pharmacother. 15, 316–326. 10.1177/0976500X241266072

[B46] Szklarczyk D. Kirsch R. Koutrouli M. Nastou K. Mehryary F. Hachilif R. (2023). The STRING database in 2023: Protein–Protein association networks and functional enrichment analyses for any sequenced genome of interest. Nucleic Acids Res. 51, D638–D646. 10.1093/nar/gkac1000 36370105 PMC9825434

[B47] Tang Z. Kang B. Li C. Chen T. Zhang Z. (2019). GEPIA2: an enhanced web server for large-scale expression profiling and interactive analysis. Nucleic Acids Res. 47, W556–W560. 10.1093/nar/gkz430 31114875 PMC6602440

[B48] Tastan Bishop Ö. Musyoka T. M. Barozi V. (2022). Allostery and missense mutations as intermittently linked promising aspects of modern computational drug discovery. J. Mol. Biol. 434, 167610. 10.1016/j.jmb.2022.167610 35490897

[B49] Venselaar H. te Beek T. A. Kuipers R. K. Hekkelman M. L. Vriend G. (2010). Protein structure analysis of mutations causing inheritable diseases. An e-Science approach with life scientist friendly interfaces. BMC Bioinforma. 11, 548. 10.1186/1471-2105-11-548 21059217 PMC2992548

[B50] Wang D. Liu D. Yuchi J. He F. Jiang Y. Cai S. (2021). MusiteDeep: a deep-learning based webserver for protein post-translational modification site prediction and visualization. Nucleic Acids Res. 48, W140–W146. 10.1093/NAR/GKAA275 32324217 PMC7319475

[B51] Wang W. Zhao R. Wang Y. Pan L. Luan F. Fu G. (2025). PLK1 in cancer therapy: a comprehensive review of immunomodulatory mechanisms and therapeutic opportunities. Front. Immunol. 16, 1602752. 10.3389/fimmu.2025.1602752 40612941 PMC12222146

[B52] Wierer M. Verde G. Pisano P. Molina H. Font-Mateu J. DiCroce L. (2013). PLK1 signaling in breast cancer cells cooperates with estrogen receptor-dependent gene transcription. Cell Rep. 3, 2021–2032. 10.1016/j.celrep.2013.05.024 23770244

[B53] Worth C. L. Preissner R. Blundell T. L. (2011). SDM--a server for predicting effects of mutations on protein stability and malfunction. Nucleic Acids Res. 39, W215–W222. 10.1093/nar/gkr363 21593128 PMC3125769

[B54] Yadav A. K. Singh T. R. (2021). Novel structural and functional impact of damaging single nucleotide polymorphisms (SNPs) on human SMYD2 protein using computational approaches. Meta Gene 28, 100871. 10.1016/j.mgene.2021.100871

[B55] Zhang Y. (2005). TM-align: a protein structure alignment algorithm based on the TM-score. Nucleic Acids Res. 33, 2302–2309. 10.1093/nar/gki524 15849316 PMC1084323

