# Supplementary material for: Functional and structural impacts of oncogenic missense variants on human polo-like kinase 1 protein
Source: Front Bioinform. 2025 Dec 2;5:1680578. doi: 10.3389/fbinf.2025.1680578 (PMC12704984; doi:10.3389/fbinf.2025.1680578)
Supplement: Supplementary file 1 [file Supplementaryfile1.docx]

**Supplementary Figures**


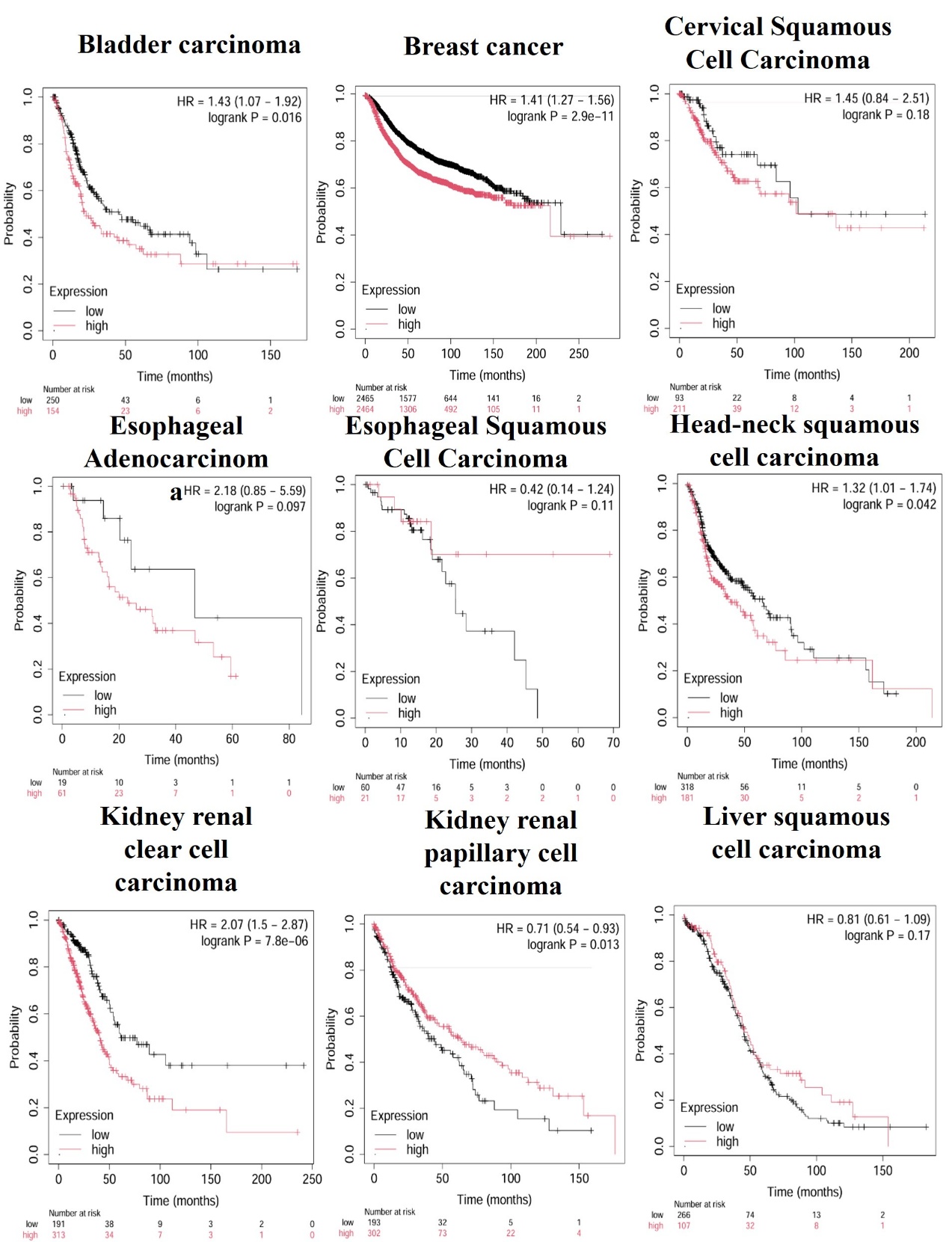


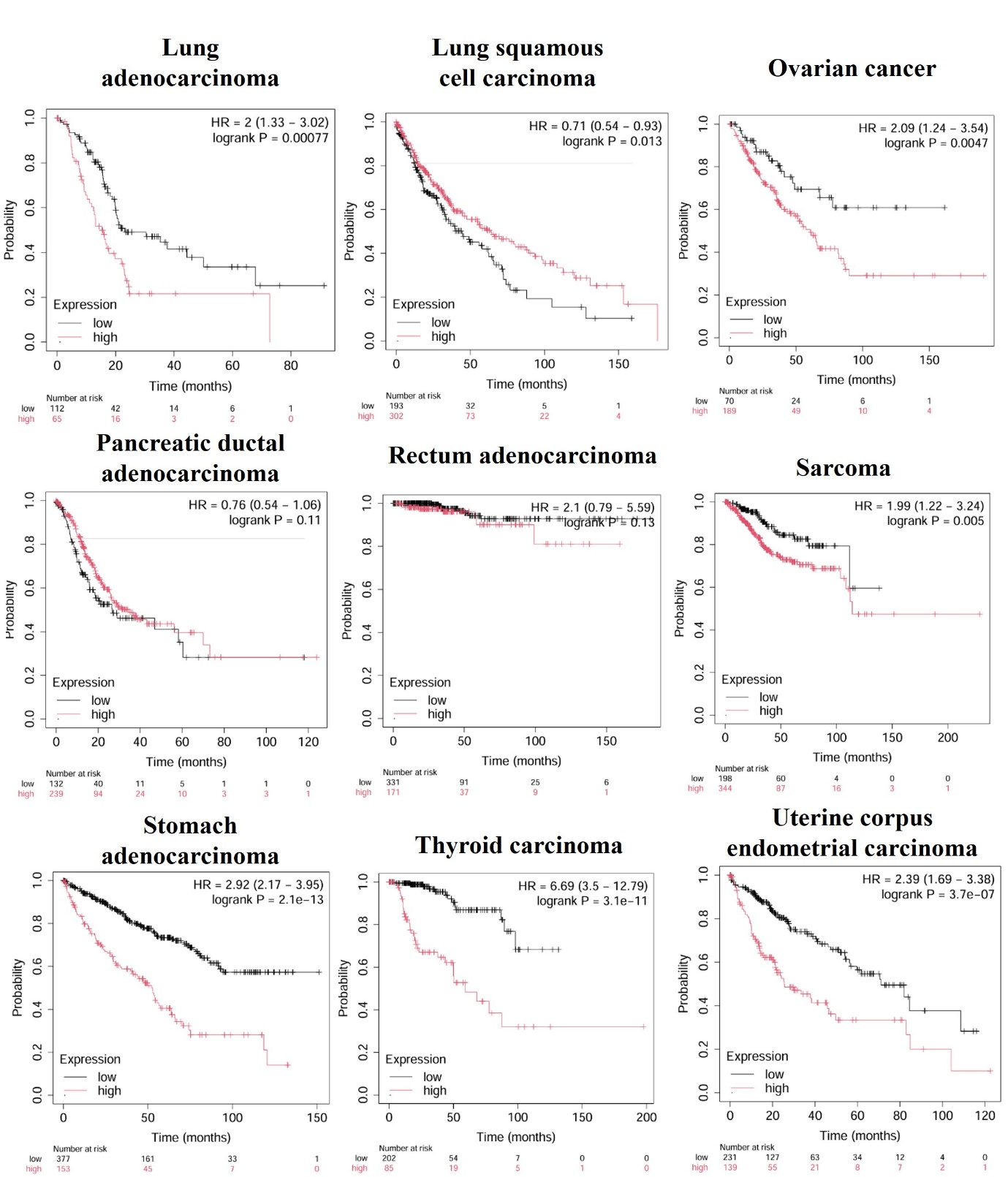


**Supplementary Figure 1**: Overall survival of PLK1 expression across all type of cancers.


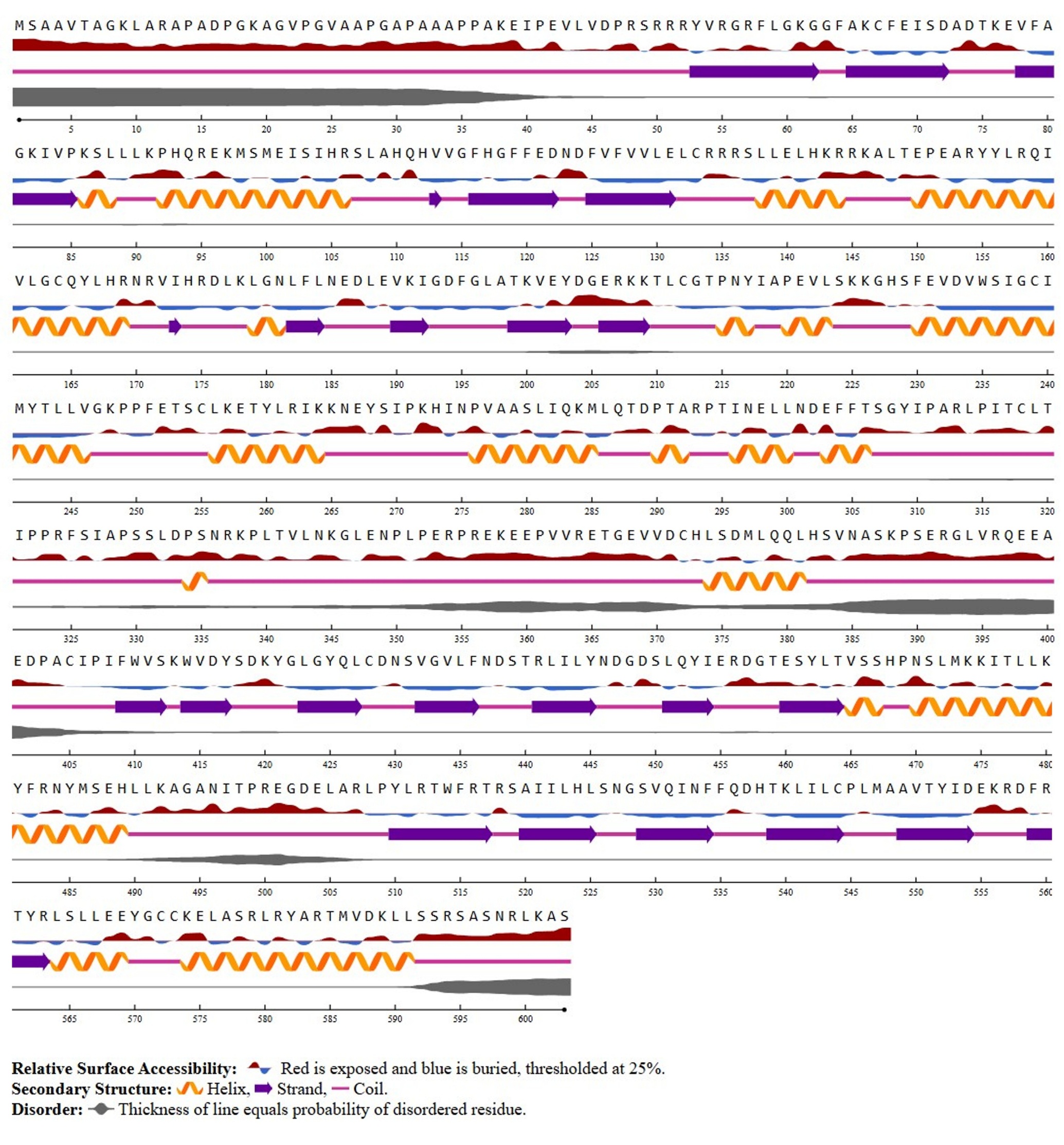


**Supplementary Figure 2**: Graphical representation of PLK1 secondary structure, relative surface accessibility and disorder predicted through NetSurf.


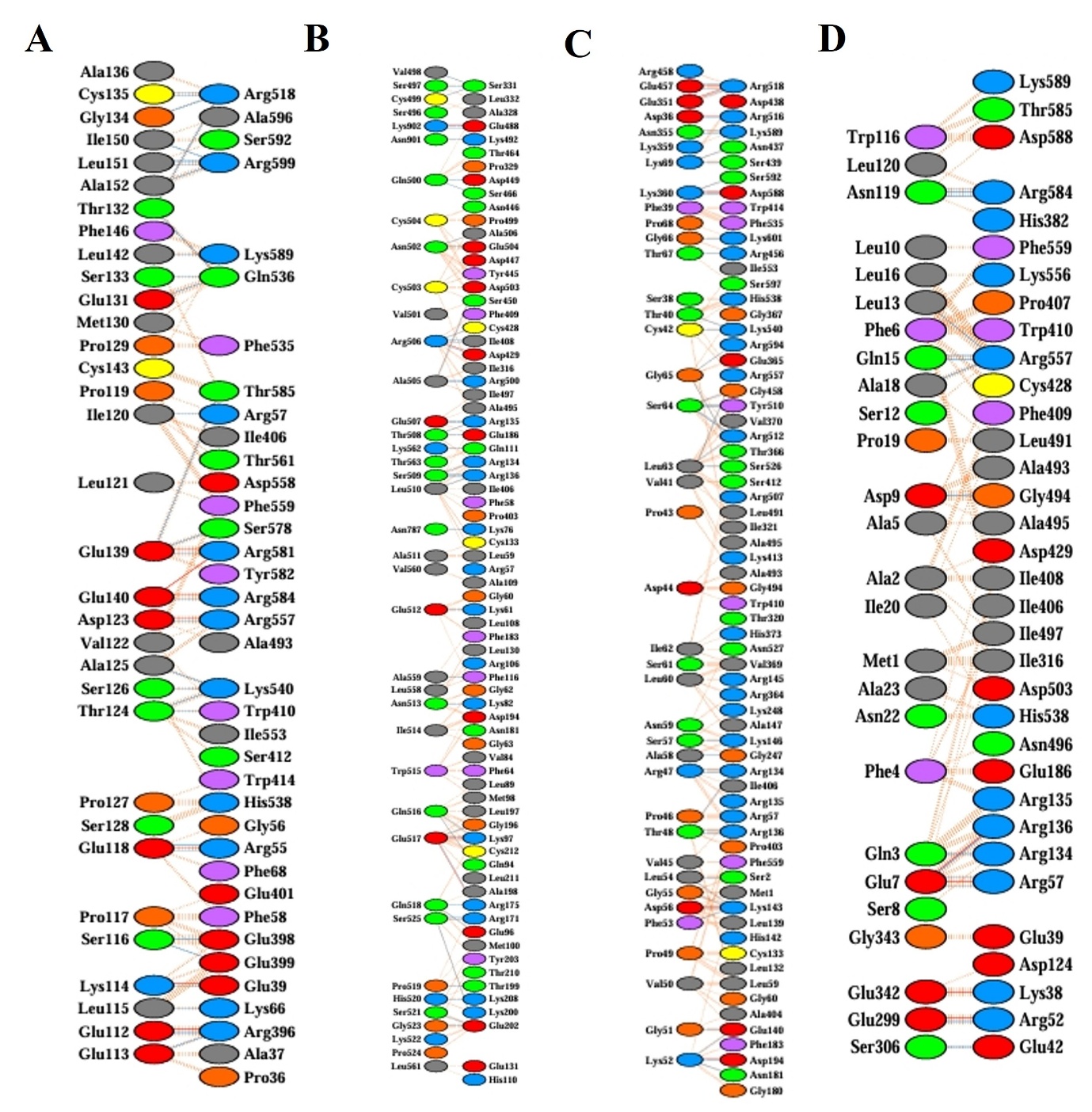


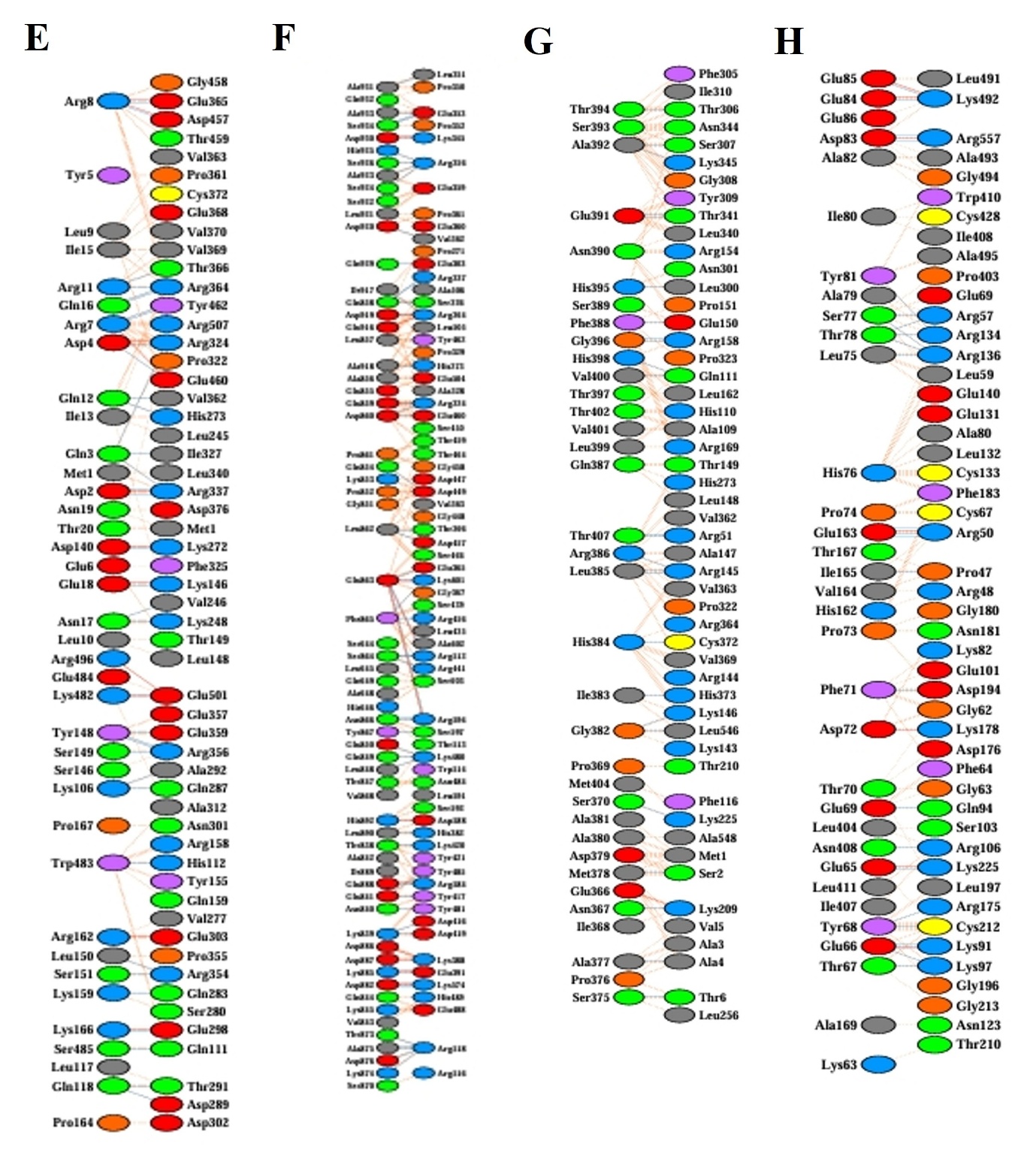


**Supplementary Figure 3**: Pictorial representation of PPI docking visualized through PDBsum server, where red color represents salt bridges, blue color represents hydrogen bonds and orange color represents non-bonded contacts, A-PLK1-CCNB1, B-PLK1-BUB1B, C-PLK1-CDC25C, D-PLK1-CDC20, E-PLK1-FZR1, F-PLK1-ERCC6L, G-PLK1-BORA and H-PLK1-BUB1
